# Exploring the Removal of Thiocarbonylthio Chain Ends from Poly(styrene-*alt*-maleic anhydride) Copolymers

**DOI:** 10.1021/acs.macromol.5c01887

**Published:** 2025-09-03

**Authors:** Michael-Phillip Smith, Lauren E. Ball, Ilanie Wessels, Bert Klumperman

**Affiliations:** Department of Chemistry and Polymer Science, 26697 Stellenbosch University, Matieland 7602, South Africa

## Abstract

Poly­(styrene-*alt*-maleic anhydride) (SMAnh), an alternating copolymer composed of electron-rich styrene (STY) and electron-deficient maleic anhydride (MAnh) comonomers, was synthesized via reversible addition–fragmentation chain transfer (RAFT)-mediated polymerization, using either a trithiocarbonate, dithiobenzoate, or dithiocarbamate chain transfer agent (CTA). SMAnh copolymers with different terminal repeat units (MAnh vs STY) were subjected to either radical-induced reduction or thermolysis to facilitate the transformation of the thiocarbonylthio functional chain end. The chemical composition of the ω-chain end and the solvency conditions employed during each end-group removal process were found to significantly influence the rate and extent of removal/transformation of the thiocarbonylthio functional group. MAnh-functional ω-chain ends enhanced the lability of the thiocarbonylthio group for all end-group removal strategies assessed, suggesting that electron-deficient chain ends facilitate higher efficiency removal of thiocarbonylthio functional groups. Additionally, 3,5-dimethyl pyrazole dithiocarbamate chain ends were reduced or thermolyzed faster and to a higher degree than trithiocarbonate- or dithiobenzoate-functional chain ends.

## Graphical abstract

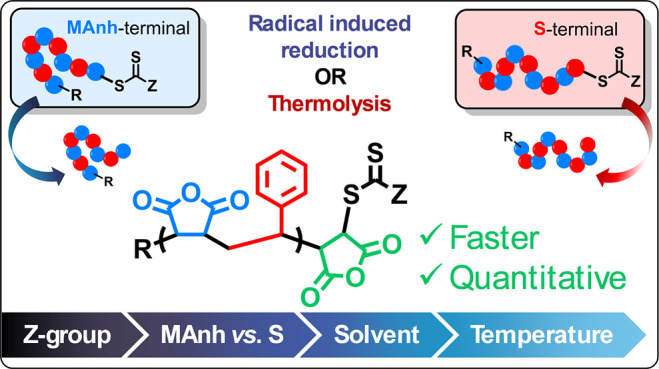

## Introduction

Controlled radical polymerization (CRP) techniques, such as reversible addition–fragmentation chain transfer (RAFT)-mediated polymerization, enable the synthesis of polymers with narrow molecular weight distributions and functional chain ends.^1^ RAFT-mediated polymerization utilizes chain transfer agents (CTAs) to reversibly deactivate the propagating polymer chain end, thereby controlling polymer growth. The thiocarbonylthio group can be removed postpolymerization if it is not required as a functional handle for further modification (e.g., a macro-CTA required for block copolymerization) or if its presence would cause side reactions upon decomposition in subsequent reactions or during storage.^2,3^

The propagation rate coefficient (*k*_p_) of a monomer is largely controlled by its electronic structure, which in turn is modulated by the nature of substituents (aromatic, aliphatic, or heteroatom-based).^4,5^ These substituents modify the electron density of the vinyl bond (via inductive effects, resonance, and electron-withdrawing effects), thereby influencing monomer reactivity with comonomers or CTAs. The monomer’s electronic structure may also impact the removal of the thiocarbonylthio group through its influence on the electronic environment of the C–S bond at the ω-chain end.^6^ Transformation of the ω-chain end can proceed via different mechanisms, such as radical-induced reduction^7–10^ and thermally induced processes.^11–13^ To investigate the influence of monomer electronic structure, CTA type, and end-group removal conditions (thermal and radical-mediated reduction as well as solvency conditions) on the transformation of the thiocarbonylthio group, poly­(styrene-*alt*-maleic anhydride) (SMAnh) was selected as a model copolymer due to the disparate electronics of its constituent comonomers (Figure 1).^2,14–21^ The copolymer is often utilized as a starting material for multistep synthetic protocols, as the MAnh repeat units are versatile functional handles, therefore affording SMAnh derivatives ubiquitous application in membrane protein isolation,^2,14–18^ biomedicine,^19–21^ and more. The presence of thiocarbonylthio groups and their potential slow degradation can cause problems related to color, odor, and toxicity.^22^ For synthetic protocols that include postpolymerization modification of the MAnh repeat units (generally using nucleophiles such as primary amines),^2,14^ aminolysis of the thiocarbonylthio group is possible as a competing reaction, which increases the prevalence of free thiols or disulfide coupling of copolymer chains.^23^ This introduces a reactive chain end that can partake in side reactions during subsequent synthetic steps or alter the molecular weight distribution of the copolymer. Thus, it is highly beneficial to optimize a thiocarbonylthio removal protocol for SMAnh, which eliminates potential interference of the end group in subsequent synthetic steps.

**Figure 1:**
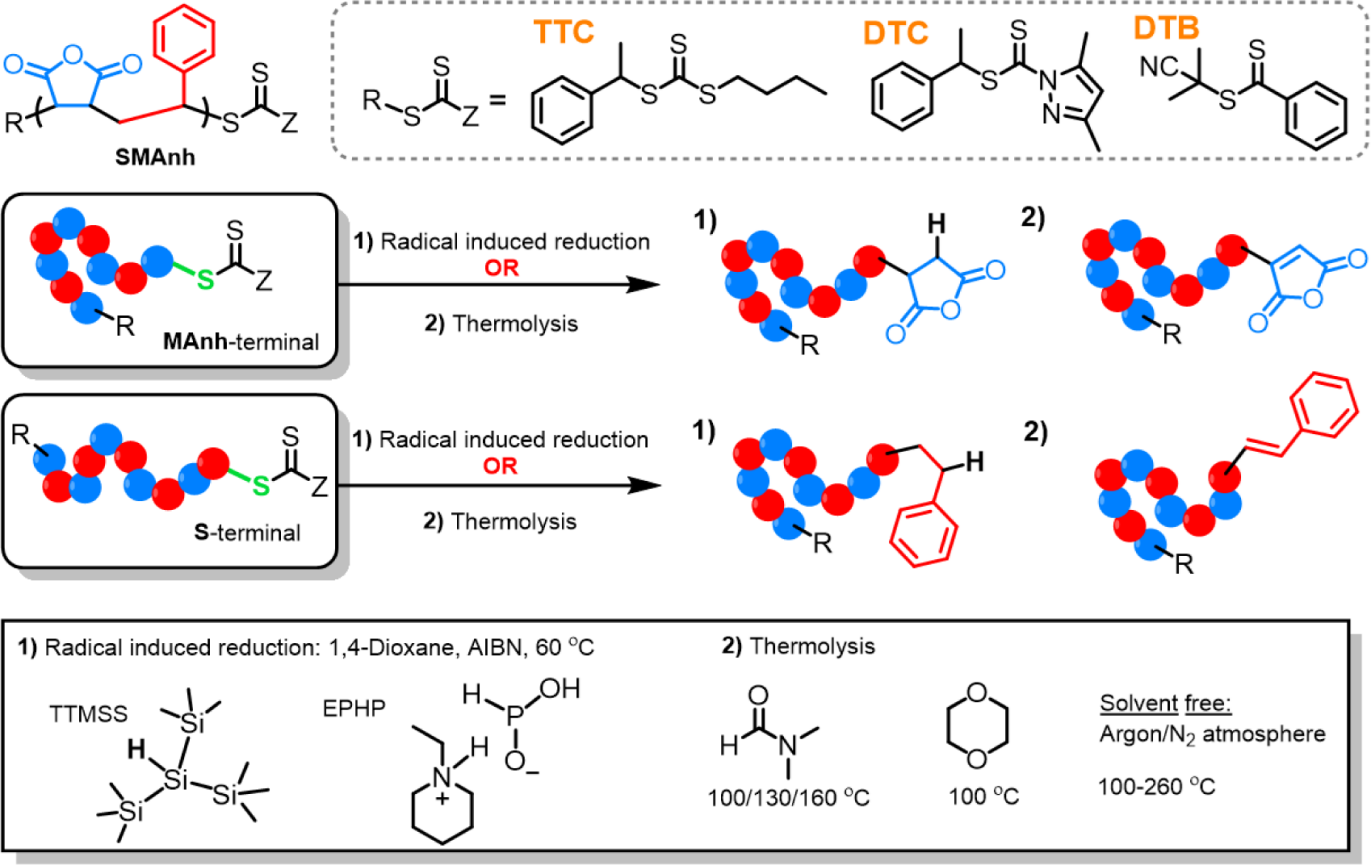
Removal of the thiocarbonylthio group from MAnh- or STY-functional SMAnh chain ends via radical-induced reduction or thermolysis.

SMAnh is an alternating copolymer, comprising styrene (STY), a strong electron donor, and maleic anhydride (MAnh), an acceptor.^24^ The electronic properties arise from the aromatic ring and the cyclic anhydride substituents, respectively, resulting in polymer chains terminated with either highly stabilized (STY) or unstabilized (MAnh) radicals. In this study, SMAnh terminated with either STY or MAnh repeat units was synthesized using CTAs with different reactivities, namely a trithiocarbonate (TTC), dithiobenzoate (DTB), and dithiocarbamate (DTC), which have been shown previously to afford SMAnh with low dispersity and high chain-end fidelity.^25^ Xanthates were excluded as they do not provide adequate control over the RAFT-mediated synthesis of SMAnh.^25,26^ These copolymers underwent end-group removal via radical-induced reduction or thermolysis, whereby the effect of the ω-chain-end chemical composition and solvency conditions on the efficiency of end-group removal was assessed. Subsequently, recommendations for the most appropriate terminal repeat unit, CTA type, solvent type, and end-group removal strategy were made in order to afford well-defined SMAnh with unreactive chain ends and an unchanged backbone.

## Materials and Methods

### Materials

Unless stated otherwise, all chemicals were purchased from Merck Life Sciences. Styrene (≥99%, *tert*-butylcatechol stabilized) was eluted from an aluminum oxide (activated basic, Brockmann I) column prior to use. Maleic anhydride (99%) and azobis­(isobutyronitrile) (AIBN, 98%) were recrystallized from dry chloroform or methanol, respectively, and thereafter dried under vacuum overnight. 1,3,5-Trioxane (99%) was purified via sublimation. Magnesium shavings were dried in an oven prior to reaction; tetrahydrofuran was distilled with sodium and benzophenone. Ethyl acetate (90%) was distilled prior to use. 1,4-Dioxane (anhydrous), CDCl_3_ (deuteration degree min. 99.8% for NMR spectroscopy, MagniSolv), tris­(trimethylsilyl)­silane (TTMSS, 97%), triethylamine (>99.5%), 1-ethylpiperidine hypophosphite (EPHP, 95%), *N*,*N*-dimethylformamide (DMF, anhydrous), potassium hydroxide (90%, flakes), carbon disulfide (Kimix, 99%), (CD_3_)_2_CO (deuteration degree min. 99.9% for NMR spectroscopy, MagniSolv), 3,5-dimethylpyrazole (99%), 1-bromoethylbenzene (97%), and 1-butanethiol (99%) were used as received.

### Polymer Synthesis

All CTAs were synthesized according to the literature, where similar yields and purities as previously reported were obtained.^25^ The TTC-mediated synthesis of SMAnh is described as a representative copolymerization with experimental parameters for the remaining copolymerizations summarized in Table 1. For the synthesis of STY-terminal SMAnh (TTC-S), styrene (6.4 g, 61 mmol), maleic anhydride (5.0 g, 51 mmol), TTC (0.92 g, 3.4 mmol), AIBN (56 mg, 0.34 mmol), 1,3,5-trioxane (68 mg, 0.75 mmol), and 1,4-dioxane (38 mL) were added to a 3-neck round-bottom flask fitted with a bubbler, magnetic stirrer bar, and rubber septum. The reaction mixture was sparged with dry argon for 45 min, a kinetic sample (0.1 mL) was withdrawn with a degassed syringe, and the reaction flask was subsequently immersed in an oil bath preheated at 60 °C for 24 h. The reaction mixture was cooled, a kinetic sample was withdrawn (0.1 mL), and the copolymer was isolated via precipitation in pentane (0.5 L). The precipitate was isolated via vacuum filtration and dried under a vacuum overnight. All copolymers were characterized using SEC, ^1^H NMR, ATR-FTIR, and UV–vis spectroscopy.

**Table 1:** Experimental Parameters for STY-MAnh Copolymerizations

Sample	DP (S, MAnh)^a^	Time^b^(h)
TTC-S	18, 15	24
TTC-M	15, 17	24
DTC-S	18, 15	22
DTC-M	15, 19	7 *
DTB-S	19, 15	24
DTB-M	15, 18	24

a DP­(STY) = [STY]/[CTA] or DP­(MAnh) = [MAnh]/[CTA].

b All copolymerizations were conducted at 30% (w/v), 60 °C, using a [CTA]:[AIBN] ratio of 1:0.1. The reaction marked with an asterisk is stopped at a significantly lower reaction time due to the lability of the thiocarbonylthio group at high monomer conversion and in the presence of excess MAnh.^3^

### Radical-Induced Reduction

All thiocarbonylthio end-group removal reactions conducted via radical-induced reduction employed either TTMSS or EPHP as the proton donors and AIBN as the exogenous radical source. Considering the radical-induced reduction of TTC-S chain ends as a representative reaction, TTC-STY (1.5 g, 0.42 mmol), a proton donor (TTMSS or EPHP, 1.3 mmol, 3 equiv with respect to TTC-S chain ends), AIBN (69 mg, 0.42 mmol), and 1,4-dioxane (6.5 mL) were added to a 3-neck round-bottom flask fitted with a bubbler, magnetic stirrer bar, and rubber septum. The solution was sparged with dry argon for 0.5 h, and the flask was subsequently immersed in an oil bath preheated to 60 °C. Kinetic samples (0.8 mL) were withdrawn with a degassed syringe at specified time intervals, and the copolymer was purified via precipitation in pentane (40 mL). The precipitate was isolated via centrifugation; the copolymer pellets were dried under vacuum overnight and subsequently dissolved in 1,4-dioxane or THF (5% AcOH) at specified concentrations to quantify the removal of the thiocarbonylthio group via UV–vis spectroscopy and SEC, respectively.

### Solvated Thermolysis

Copolymer samples were solubilized in dioxane or DMF at 20% (w/v) in a 50 mL round-bottomed flask sealed with a rubber septum. The flask was thereafter immersed in a preheated oil bath (100, 130, or 160 °C). All samples were collected at the indicated time intervals via a degassed syringe. On collection, samples were immediately precipitated into diethyl ether and centrifuged. This process of centrifugation and washing (with diethyl ether) was repeated thrice. Kinetic samples were dried *in vacuo* at room temperature overnight. For samples that underwent thermolytic cleavage, the isolated styrene-terminated copolymers were an off-white color, while maleic anhydride-terminated copolymers were dark brown. In contrast, copolymers that underwent thermolytic cleavage in dioxane did not discolor and were instead lighter derivatives of the original color, i.e., DTB copolymers formed a lighter shade of pink, etc.

### Solvent-Free Thermolysis

Thermogravimetric analyses were conducted on a TGA Q500 instrument under a flow of N_2_ gas with a flow rate of 50 mL·min^–1^. Samples were loaded onto aluminum pans and subjected to a temperature ramp from 20 to 600 °C (10.00 °C·min^–1^). The thiocarbonylthio removal temperature was identified as the first significant loss of mass following residual solvent removal at ca. 150 °C. Solvent-free thermolysis was then conducted in bulk, whereby each copolymer sample (80–100 mg) was subjected to its corresponding onset end-group removal temperature (identified via TGA) for 150 min. All samples were subsequently purified via dissolution in acetone (about 1 mL) and precipitation in pentane (about 35 mL). This process was repeated three times, and the copolymers were subsequently dried under vacuum overnight at 50 °C prior to characterization via UV–vis spectroscopy and SEC.

## Results and Discussion

### Polymer Synthesis

SMAnh copolymers were synthesized via RAFT-mediated copolymerization using three different CTAs with dissimilar reactivity (Figure 1), which are known to facilitate the synthesis of well-defined SMAnh-type copolymers.^25^ Notwithstanding the experimental parameters employed for the removal of the thiocarbonylthio group (i.e., removal technique, solvent, temperature, etc.), the lability of the C–S bond (indicated in green, Figure 1) is influenced by the electronics of the ω-chain end, which is in turn governed by the Z-group employed and the nature of the attached polymer.^3^ To investigate the interplay between the nature of the Z-group and polymer, SMAnh with either a MAnh- or STY-functional ω-chain end was synthesized according to a previously reported protocol.^3^

For each copolymerization, nearly quantitative monomer conversion was achieved such that a slightly higher DP was obtained for the comonomer in excess (*f*_0_^S/MAnh^ > 0.5) in the comonomer feed (Table 2). The copolymerization of STY and MAnh has a strongly alternating character; therefore, a predominantly alternating block of SMAnh was obtained followed by one MAnh repeat unit (in the case of *f*_0_^STY^ < 0.5) or approximately 2 styrene repeat units (in the case of *f*_0_^STY^ > 0.5). The synthesis of either MAnh- or STY-functional ω-chain ends was confirmed via ^1^H NMR spectroscopy, whereby the protons of the terminal repeat unit (H_A_ and H_B_, Figure 2) exhibit characteristic chemical shifts between 3.5 and 5.5 ppm.^3,27^ SEC analyses indicated that SMAnh copolymers with low *Đ* (1.09–1.22) had been synthesized with good correlation between *M*_*n*_^theo^ and *M*_*n*_^SEC^ (Table 2 and Figures S1–S3). These copolymers subsequently underwent thiocarbonylthio group removal, either via radical-induced reduction or thermolysis, to assess the effect of varying experimental parameters and the chemical composition of the ω-chain end on end-group removal efficiency.

**Table 2:** Monomer Conversion and Molecular Weight Data for All of the SMAnh Copolymers

Sample^a^	α (%S, %MAnh)^b^	DP (S, MAnh)^c^	*M*_n_^theo^ (g/mol)^d^	*M*_n_^SEC^ (g/mol)^e^	*Đ* ^e^
TTC-S	96, 100	17, 15	3 500	4 000	1.11
TTC-M	100, 92	15, 15	3 300	3 600	1.11
DTC-S	96, 100	17, 15	3 500	3 700	1.13
DTC-M	100, 79	15, 15	3 300	2 700	1.22
DTB-S	71, 82	14, 12	2 900	4 100	1.09
DTB-M	85, 73	12, 13	2 800	3 800	1.09

a TTC, DTC, and DTB indicate the CTA used and S or M indicate styrene or maleic anhydride terminal repeat units, respectively.

b Monomer conversion was determined via ^1^H NMR spectroscopy using 1,3,5-trioxane as an internal standard and eq S1.

c The degree of polymerization was calculated using α and the targeted DPs in Table 1.

d Theoretical *M*_*n*_ calculated using α and eq S2.

e It was determined via SEC using THF (5% AcOH) as the mobile phase and PSTY calibration standards.

**Figure 2:**
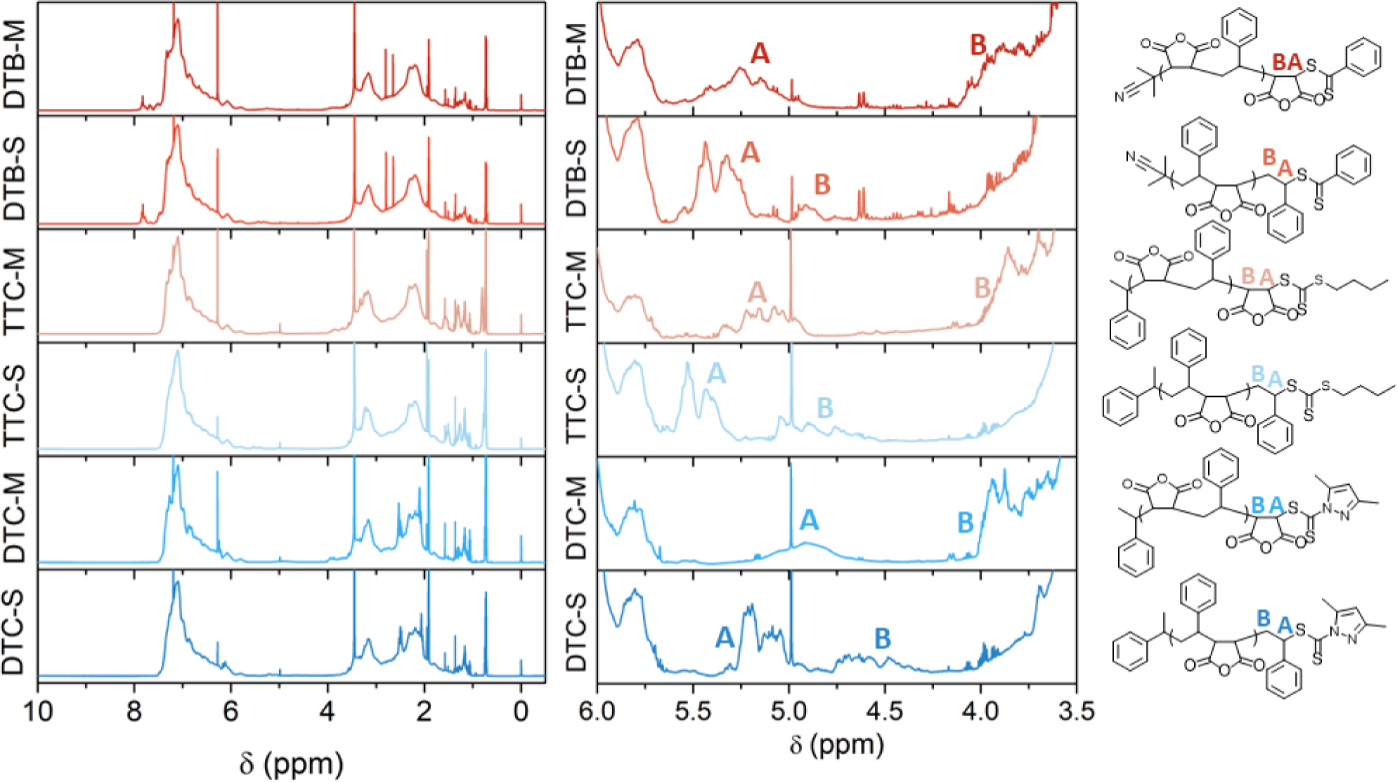
^1^H NMR spectra of all SMAnh copolymers in acetone-*d*_6_. The terminal repeat unit protons are labeled H_A_ and H_B_. Signals at 3.5, 1.9, and 0.8 ppm represent residual 1,4-dioxane, acetone, and pentane respectively, while signals at 6.3 and 5.0 ppm represent residual maleic acid and 1,3,5-trioxane, respectively.

### Radical-Induced Reduction

The radical-induced reduction process exploits the ability of thiocarbonylthio functional compounds to partake in facile addition–fragmentation reactions with radicals. Briefly, a radical (derived from an exogenous source such as AIBN) adds to the thiocarbonylthio functional group of the SMAnh macro-CTA, resulting in the formation of an intermediate radical (e.g., TTC-M-AIBN in Figure 3C). This fragments to yield a new thiocarbonylthio compound and a propagating radical species, which then ideally reacts with a proton donor (such as TTMSS or EPHP) to yield SMAnh with a hydrogenated ω-chain end. TTMSS and EPHP have historically exhibited the highest activity as proton donors, facilitating the rapid reduction of both dithioester-, dithiocarbamate-, and trithiocarbonate-functional chain ends, where radical production was achieved either via thermal decomposition of an azoinitiator or more recently via photoinduced β-fragmentation of the thiocarbonylthio group.^9,28^ In the present study, AIBN was employed as the radical source in conjunction with TTMSS or EPHP, using a [macro-CTA]:[AIBN]:[TTMSS/EPHP] ratio of 1:1:3.

**Figure 3:**
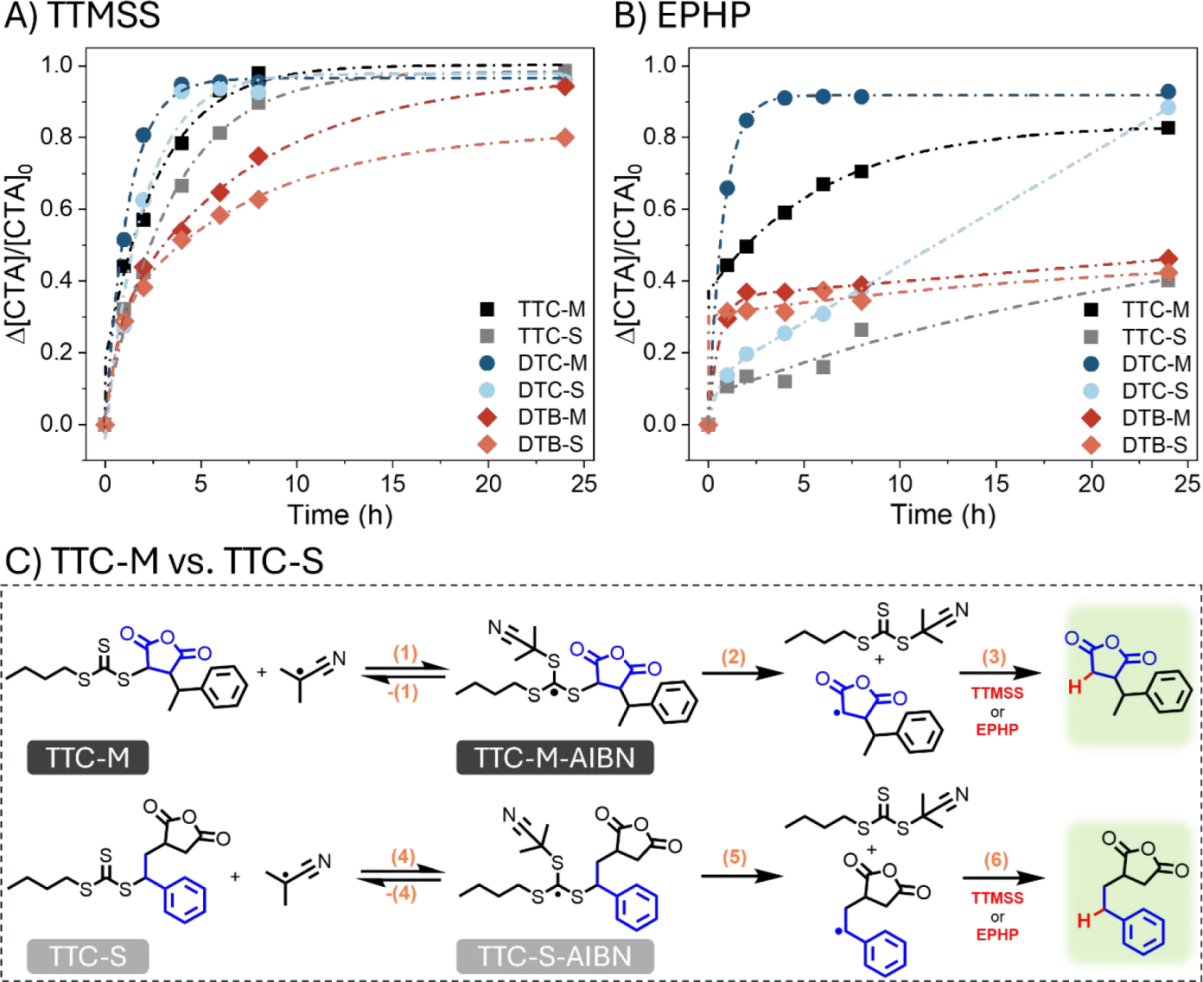
Kinetic analysis of the radical-induced reduction of SMAnh thiocarbonylthio chain ends using TTMSS (A) or EPHP (B) as proton donors. The proposed reaction mechanism, associated with the addition of AIBN radicals to TTC-M or TTC-S (Δ*G*_add_), fragmentation of the resulting intermediate radical TTC-M-AIBN or TTC-S-AIBN (Δ*G*_frag_) and the abstraction of a proton from either proton donor (Δ*G*_abs._), was assessed computationally and summarized in a scheme (C).

For DTC-M and DTC-S, the data are nearly quantitative (93–95% as determined via UV–vis spectroscopy, Figure S4) removal of the thiocarbonylthio moiety was observed within 4 h at 60 °C in 1,4-dioxane, using TTMSS as the H-donor (Figure 3A). TTC-M exhibited 98% end-group removal within 8 h under the same conditions, whereas TTC-S required up to 24 h of TTMSS-mediated reduction to achieve 99% desulfurization (Figure S5). Of the copolymers assessed, the TTMSS-mediated reduction of DTB-SMAnh ω-chain ends was the slowest, where DTB-M could be fully reduced (ca. 94%) within 24 h, but DTB-S still retained 20% thiocarbonylthio chain ends at 24 h (Figure S6). There appears to be a clear influence of the Z-group and terminal repeat unit composition on end-group removal efficiency. The Z-group, which stabilizes the intermediate radical more effectively (DTB > TTC > DTC), results in slower fragmentation,^29^ which in turn results in a lower concentration of SMAnh-derived radicals. Furthermore, MAnh-based radicals have a higher reactivity than stabilized STY-based radicals and thus undergo reduction at a faster rate than STY-functional ω-chain ends. The slower reduction of the STY-functional chain ends is unsurprising. Polystyrene is generally a poor homolytic leaving group and the benzylic radicals formed upon fragmentation of PSTY-based intermediate radicals are stable and therefore tend to undergo competitive termination via combination or hydrogen abstraction.^9^ This is a persistent obstacle during the desulfurization of RAFT-synthesized polymers. However, it is unlikely that termination by combination occurs for SMAnh-based radicals (STY- or MAnh-functional chain ends) under the reducing conditions applied, as no bimodality was observed in the SEC traces for all SMAnh copolymers that underwent TTMSS/EPHP-mediated reduction (Figures S1–S3). For all copolymers, SEC analysis showed that the radical-induced reduction process had minimal effect on the molecular weight distribution, but UV detection (at 320 nm) indicated that desulfurization of the ω-chain end was complete after 24 h (>99.6% removal), with the exception of DTB-S (79% removal).

EPHP is an interesting alternative to TTMSS for the desulfurization of SMAnh-type copolymers, as the byproducts formed from the hypophosphite postreduction are water-soluble and can be removed via dialysis or a simple wash.^9^ If the hydrolysis of the MAnh repeat units is undesirable, then this purification method is not recommended. However, this protocol might be particularly valuable in cases where the hydrolyzed derivative of the SMAnh backbone is desired. For example, this protocol might be valuable for researchers who employ RAFT-synthesized poly­(styrene-*alt*-maleic acid) (SMA)-type copolymers as macro-detergents for the solubilization of membrane proteins.^2,14,30^

Therefore, equivalent radical-induced reduction experiments were conducted using EPHP as the H-donor. Interestingly, upon the addition of EPHP to a solution of each SMAnh macro-CTA, an orange-brown discoloration occurred (Figure S7A). It is unlikely that this discoloration corresponds to functionalization or removal of the thiocarbonylthio moiety as the π–π* absorbance band for the ω-chain end chromophore does not shift, and furthermore, EPHP-mediated reduction generally resulted in significantly slower and incomplete chain-end removal (Figure 3B). Upon inspection of the ATR-FTIR spectra for kinetic samples withdrawn during the reduction process, additional stretching frequencies that are uncharacteristic of SMAnh were observed (Figure S7A). The characteristic asymmetric and symmetric carbonyl stretching frequencies at 1849 and 1770 cm^–1^ respectively, had decreased in intensity, and an additional uncharacteristic stretching frequency had appeared at 1721 cm^–1^. These uncharacteristic carbonyl stretching frequencies were absent for SMAnh polymers pre- and post-TTMSS exposure (Figure S7B). It was hypothesized that EPHP could be interacting with MAnh repeat units along the backbone. EPHP and succinic anhydride were mixed in dioxane for 24 h to mimic the exposure of MAnh repeat units to EPHP during end-group removal. An O–H stretching frequency (3050–3660 cm^–1^) characteristic of EPHP disappeared upon exposure to succinic anhydride and an additional signal at 1712 cm^–1^ appeared, which is not characteristic of EPHP or succinic anhydride (Figure S7C). Therefore, it is highly plausible that some interaction between the EPHP and MAnh units occurred during the radical-induced reduction experiment. This putative interaction between EPHP and MAnh repeat units had not been disrupted upon purification of the kinetic samples via precipitation. Nevertheless, near-quantitative reduction could be achieved for DTC-M (97%) and DTC-S (93%, Figures 3B and S4), with no significant change in the molecular weight distribution observed (Figure S2). Once again, MAnh-functional chain ends appeared to undergo reduction more efficiently than STY-functional chain ends, and furthermore, only partial reduction of DTB-functional chain ends was observed.

The effect of disparate terminal repeat units (STY vs MAnh) was also assessed computationally, as summarized for TTC-copolymers in Figure 3C. The calculated free energy of addition (Δ*G*_addition_) for AIBN-derived radicals to TTC-functional chain ends (for the formation of the intermediate radical species) was lower when bound to an MAnh unit (14.0 kcal·mol^–1^ for TTC-M-AIBN, **1**) compared to an STY unit (16.4 kcal·mol^–1^ for TTC-S-AIBN, **4**). The fragmentation of the TTC-S-AIBN intermediate radical (−11.3 kcal·mol^–1^, **5**) is more favorable compared to TTC-M-AIBN intermediate radicals (−9.9 kcal·mol^–1^, **2**), but the abstraction of a proton (from TTMSS or EPHP) by the resulting STY-based radical is less favorable (−1.8 kcal·mol^–1^, **6**) than the MAnh-based radical (−7.4 kcal·mol^–1^, **3**). This aligns well with the experimentally observed trends.

While the radical-induced reduction of thiocarbonylthio-functional SMAnh proved to be an efficient end-group removal strategy, particularly for the DTC or TTC-M copolymers, the H-donors utilized are expensive and difficult to remove completely during purification. Thermolysis potentially offers a facile thiocarbonylthio group removal strategy for SMAnh copolymers, as the process does not produce persistent byproducts and does not require the use of expensive reagents.

### Thermolysis

Thermolysis is a process that exploits the inherent thermal lability of the thiocarbonylthio functional group, generating a terminal alkene at the copolymer ω-chain end.^12,13^ There are limited studies investigating the varying thermal lability of different thiocarbonylthio groups (dithioester, dithiocarbamate, and trithiocarbonate).^11–13^ In this study, initial thermolysis experiments were conducted in *N*,*N*’-dimethylformamide (DMF). The benefits of using DMF are 2-fold, i.e., it ensures efficient solvation of SMAnh and thus homogeneous heating of the copolymer and additionally, it allows for thermolysis within a wide range of elevated temperatures (DMF bp 153 °C). Thermolysis experiments were conducted over 24 h at 100, 130, and 160 °C (Figure 4, Table S1, Figures S8 and S9) on TTC-M and TTC-S copolymers to assess the ideal temperatures required for the removal of the thiocarbonylthio group. It is noted that TTC-M reaches quantitative thiocarbonylthio group removal at all temperatures. Contrarily, TTC-S ω-chain ends were fully thermolyzed at 160 °C; however, 130 and 100 °C afforded only 92% and 67% thermolysis, respectively (Figures 4A and S8). Therefore, it can be noted that a higher temperature favors a greater degree and faster rate of thiocarbonylthio thermolysis. As with the radical-induced reduction reactions, there is a clear influence of the nature of the terminal repeat unit on thiocarbonylthio group removal efficiency, as copolymers with MAnh-functional ω-chain ends consistently undergo quantitative and rapid thermolysis irrespective of the macro-CTA type (Figure 4A). Based on NMR spectroscopy techniques (Figures S10 and S11) it was possible to assign that the thermolytic cleavage of SMAnh-S and SMAnh-M chains resulted in the formation of the expected trans-vinyl styrene chain end^13^ and the vinyl maleic anhydride chain end, respectively. The effect of terminal repeat units was further assessed computationally for TTC-copolymers in Figure 4B. The calculated free energy of thermolysis of TTC-functional chain ends was lower when bound to an MAnh repeat unit (−3.8 kcal·mol^–1^, **1**) compared to an STY repeat unit (6.1 kcal·mol^–1^ for TTC-S-cis, **2**, and 3.7 kcal·mol^–1^ for TTC-S-trans, **3**). This data align with experimentally observed trends.

**Figure 4:**
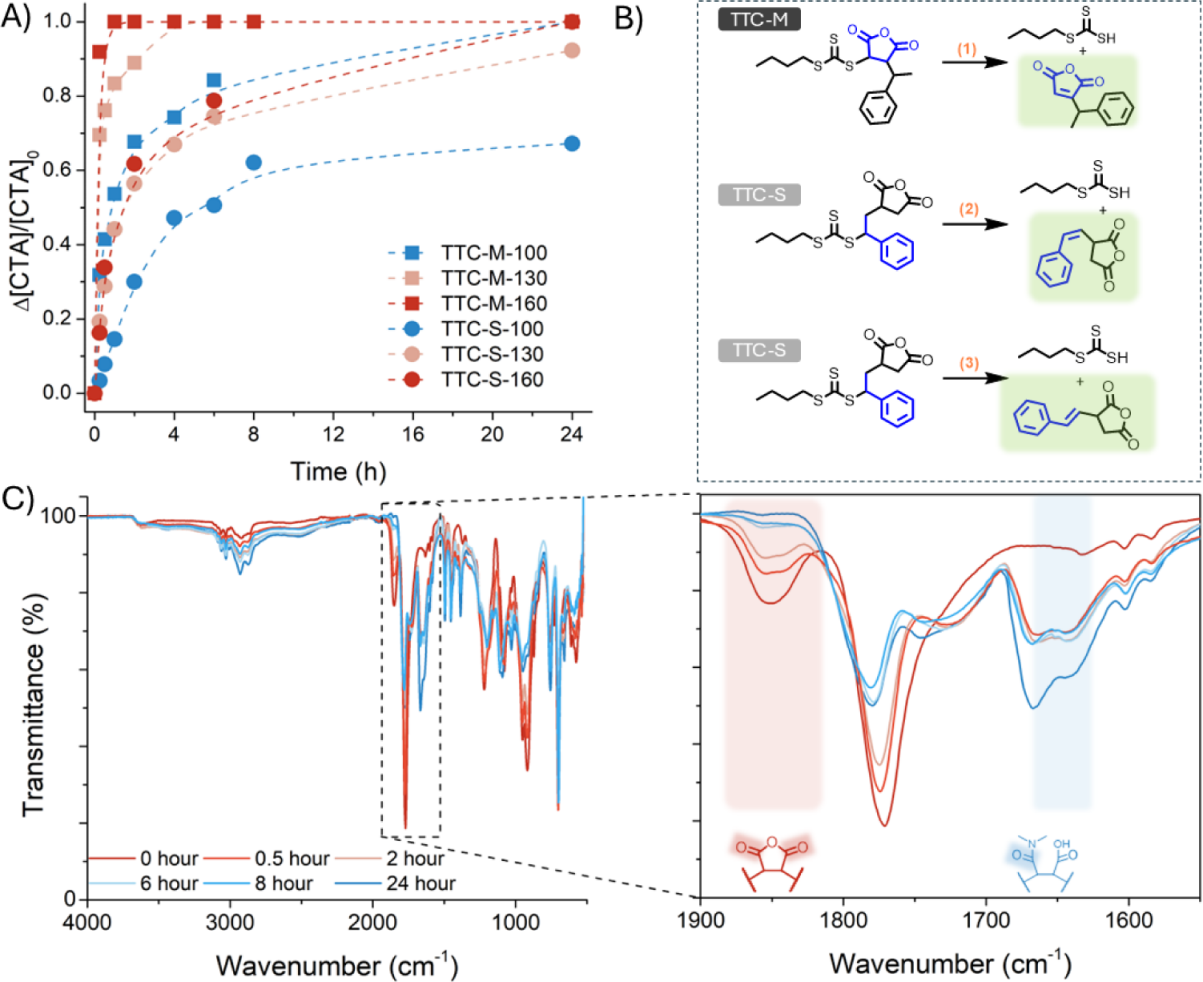
Kinetic analysis of the thermolysis of SMAnh thiocarbonylthio chain ends using DMF as the solvent. A) TTC-S and TTC-M copolymers thermolyzed at various temperatures for 24 h. B) The proposed mechanism associated with the thermolytic cleavage of TTC-S and TTC-M was assessed computationally. C) ATR-FTIR spectra indicating alteration of MAnh units along the SMAnh backbone at 160 °C (using DTB-M as an exemplary copolymer).

The reactivity of MAnh-functional chain ends during radical-induced reduction or thermolysis poses an interesting opportunity to overcome some challenges associated with the desulfurization of RAFT-synthesized polymers, particularly those that produce highly stabilized radical chain ends or exhibit chain-end thermolysis temperatures near the polymer’s degradation temperature. The low *k*_p_ of MAnh makes it an excellent candidate for sequence-controlled copolymerization, single-unit monomer insertion (SUMI) and polymer chain-end functionalization.^27,31–34^ For RAFT-synthesized polymers, which do not undergo facile desulfurization, it is plausible that the SUMI of MAnh into the macro-CTA prior to end-group removal would significantly improve the efficiency of the process, as is suggested by the efficient desulfurization of SMAnh with MAnh-functional chain ends in this study.

It was noted that thermolysis experiments conducted in DMF resulted in unusual discoloration of the copolymer, from yellow or pink (characteristic of thiocarbonylthio-functionalized SMAnh) to deep brown (as opposed to the white powder obtained from radically reduced SMAnh). In addition to altered solution properties of the thermolyzed copolymer (which did not precipitate in diethyl ether as effectively compared to the starting material), analysis of the thermolyzed copolymer’s chemical composition (via ATR-FTIR, Figure 4C) exhibited uncharacteristic signals for SMAnh. In the ATR-FTIR spectra, it was noted that there is a disappearance of the anhydride stretch at 1860 cm^–1^ over time and the appearance of an amide stretch at 1650 and 1740 cm^–1^ (Figure 4C).^35^ These anomalies were hypothesized to be a result of the modification of maleic anhydride repeat units with dimethyl amine. The degradation of DMF becomes prevalent at elevated temperatures and in the presence of moisture, resulting in the production of secondary amine contaminants, which are known to undergo nucleophilic addition to MAnh repeat units.^35^ This results in the formation of maleamic acid repeat units. The possible transformation of maleamic acid repeat units, during the thermolysis of DTB-M, was confirmed via ATR-FTIR spectroscopy (Figure 4C).^35^ To confirm that the observed changes in chemical composition were a result of repeat unit modification and not related to side reactions of the thiocarbonylthio group, SMA2000 (a commercial SMAnh copolymer synthesized via conventional radical copolymerization and lacking functional chain ends) was heated in DMF and changes in chemical composition were assessed. The same transformations were observed (via ATR-FTIR, Figure S12).

As the focus of this study is on the thiocarbonylthio end-group removal, we have not extensively investigated the scope of the side reaction in other scenarios. However, for postpolymerization modification of SMAnh with primary amines to form the corresponding maleimide repeat units, DMF has been used as the solvent at temperatures near or above 100 °C.^2,36–39^ Further investigation into the level of occurrence of the side reaction under a variety of experimental conditions is necessary to fully understand its importance beyond the currently studied end-group removal.

Although thermolytic cleavage in DMF results in thiocarbonylthio removal, dioxane was investigated as an additional organic solvent that does not yield possible degradation byproducts (Figure S13 and Table S2). Nevertheless, only thermolysis at 100 °C was assessed to ensure the absence of potential side reactions (evident via ATR-FTIR spectra, Figure S14) and due to solvent limitations (dioxane, bp 101 °C). The relative rate of thiocarbonylthio removal in DMF (more polar) or dioxane (more apolar) was assessed and is presented in Figure 5A,B, respectively. For all copolymers, desulfurization occurred at a faster rate in DMF than in dioxane. Additionally, the extent of desulfurization was highly dependent on the nature of the solvent, whereby DMF facilitated the highest efficiency thermolysis and argon the lowest efficiency thermolysis (Figure 5C, solvent-free thermolysis discussed in *vide infra*). This may be a result of the stabilization of a polar intermediate species present during thermolysis. MAnh-functional chain ends were furthermore noted to undergo thermolytic cleavage to a greater extent than STY-functional chain ends. In both solvents, DTC-M underwent near-quantitative thermolysis (100% in DMF, 95% in dioxane). In DMF, TTC-M and DTB-M exhibited quantitative thermolysis of the thiocarbonylthio group, while all copolymers with STY-functional chain ends did not. Notably, no copolymers exhibited complete thiocarbonylthio removal in dioxane (with the exception of DTC-M) and instead a mixture of thiocarbonylthio-functional and thermolyzed chain ends was attained (as evidenced via UV–vis spectroscopy). Thus, efficient thermolysis of SMAnh macro-CTA chain ends was achieved, through appropriate tuning of the chemical composition at ω-chain ends (DTC- and MAnh-functional) and the astute selection of solvent (dioxane) and thermolysis temperature (100 °C). In this way, desulfurization of the copolymer is obtained without possibly compromising the integrity of the copolymer repeat units. However, this method still employs expensive solvents with deleterious environmental impact. Therefore, the thermolysis of SMAnh copolymers was also investigated under nonsolvency conditions (argon atmosphere) toward an overall greener thiocarbonylthio removal protocol.

**Figure 5:**
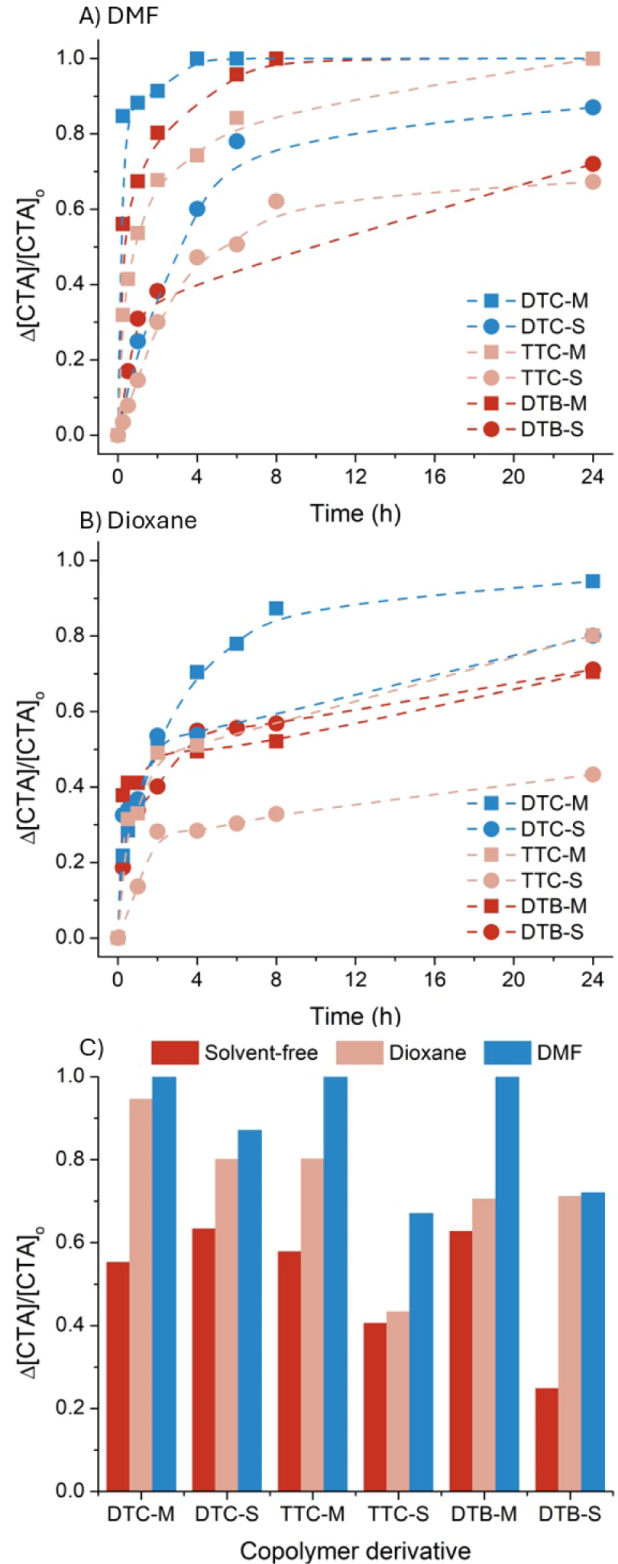
Kinetic analysis of the solvated thermolysis of SMAnh thiocarbonylthio chain ends using DMF (A) or dioxane (B) as the solvent at 100 °C. C) Summary of thermolysis experiments in all solvents/nonsolvents at 100 °C for 24 h. The corresponding ATR-FTIR spectra for the 24 h samples are presented in Figures 4C, S14, and Figure −S17 (using DTB-M as an exemplary copolymer).

### Solvent-Free Thiocarbonylthio Group Removal

To identify an operating temperature range for solvent-free thermolysis of thiocarbonylthio chain ends, all SMAnh copolymers underwent thermogravimetric analysis (TGA) as was previously done in our group for the trithiocarbonate RAFT agent.^2^ Three notable mass loss regimes were observed, the first between 100 and 180 °C, followed by 200 and 300 °C, and finally between 300 and 500 °C (Figure S15). The first mass loss (100–130 °C) likely corresponds to the removal of residual 1,4-dioxane as no decrease in the intensity of the π–π* absorbance band (300–310 nm) was observed. The second notable mass loss (170–300 °C) was ascribed to the cleavage of the thiocarbonylthio moiety as SMAnh subjected to temperatures within this range exhibited a significant decrease in π–π* absorbance band intensity, but without degradation of the backbone (Figure 6 and Table S3). The final mass loss from 300 °C corresponds to the degradation of the copolymer backbone.^40^ Some differences in the onset temperature at which thiocarbonylthio removal begins were observed, which may suggest that the different ω-chain ends have different thermal lability (Table S4). DTC-M required a considerably lower thermolysis temperature (170 °C) compared to the other polymers tested (230–260 °C), a phenomenon that has been demonstrated recently during the hydrolysis of SMAnh copolymers at significantly lower temperatures.^3^

**Figure 6:**
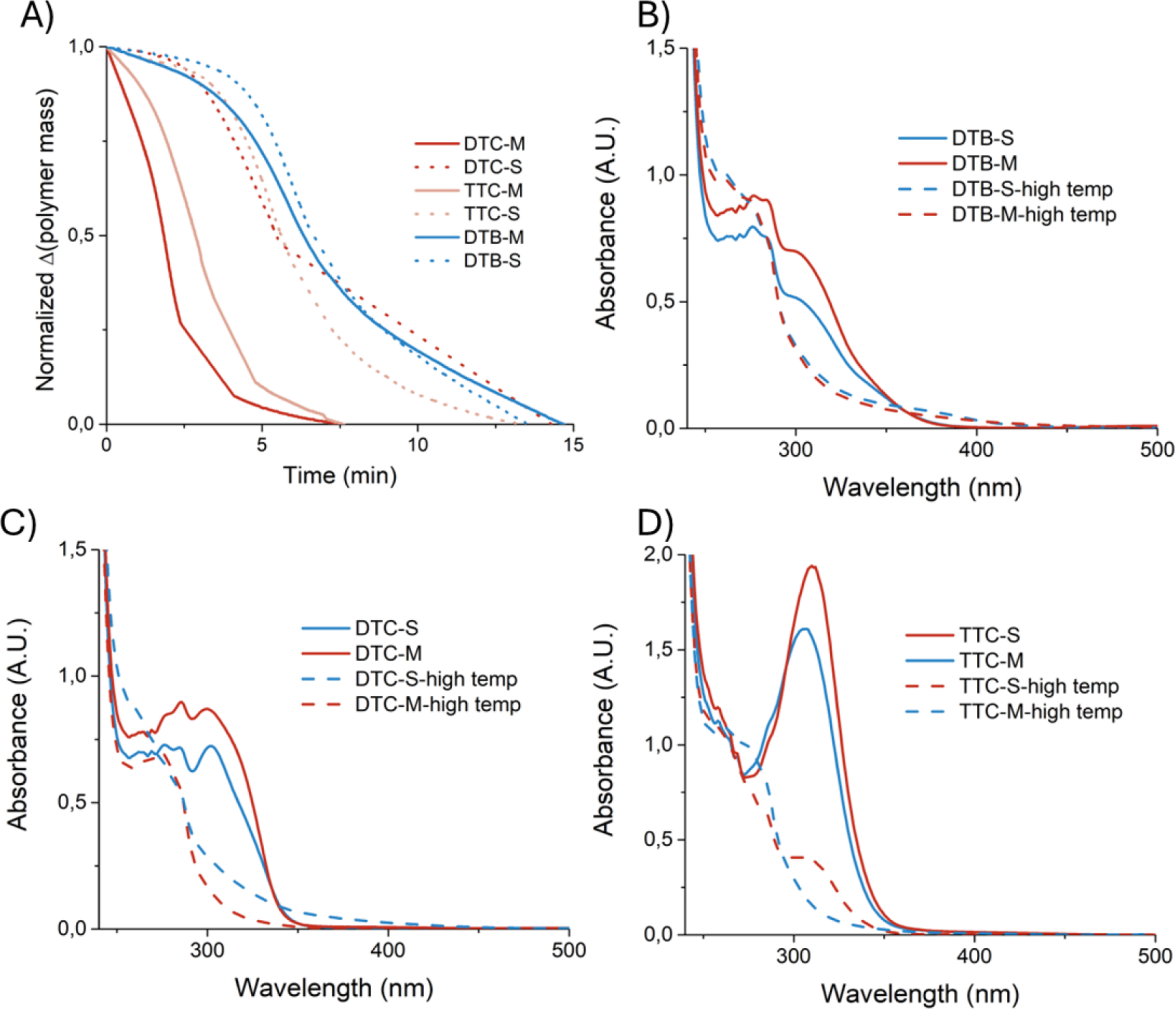
Solvent-free thermolysis thiocarbonylthio group removal. A) Percentage thiocarbonylthio present as a function of time following subjection of the polymers to the following temperature ranges: DTC-M (149.5–170 °C), DTC-S (200–250 °C), TTC-M (200–230 °C), TTC-S (180–235 °C), DTB-M (200–260 °C), and DTB-S (200–260 °C); B-D) UV–vis spectra of copolymers before thermolysis and after thermolysis at high temperatures, as indicated in Table S4.

To confirm that the mass losses observed between 170 and 260 °C corresponded to the thermolysis of the thiocarbonylthio group, each copolymer was heated to its respective thermolysis temperature (Figure 6). Disregarding the contribution of the solvent contaminant, the mass loss at approximately 20 min corresponded to 6 w/w% (DTC-M) and 8 w/w% (DTC-S) of the sample, which is consistent with the mass loss expected from the cleavage of the thiocarbonylthio moiety (Figure S16). The successful thermolysis of the ω-chain ends was confirmed via UV–vis spectroscopy (Figure 6B–D) and the absence of potential side reactions was evident via ATR-FTIR spectra (Figure S17). Continuing with the same trends observed earlier in this study, the rate of thermolytic cleavage was faster for DTC and TTC when MAnh was present as the terminal repeat unit (Figure 6A).

Following these smaller-scale reactions, the experiments were mimicked in bulk by heating the samples at 100 °C for 24 h to compare the effect of the solvent type on the thermolysis efficiency. In Figure 5C the compounded effect of ω-chain end chemical composition and solvency conditions (at 100 °C) on thiocarbonylthio group thermolysis is presented. For all copolymers, thermolytic cleavage of the thiocarbonylthio group under an argon atmosphere is lower than that observed during solvated thermolysis. This is most likely the result of inhomogeneous heating of the copolymer compared to thermolysis experiments utilizing a solvated copolymer. Nonsolvated thermolysis affords a greener synthetic protocol due to the exclusion of toxic solvents; however, energy requirements are higher due to the application of the high temperatures required for quantitative thermolysis. Of all copolymer derivatives assessed, DTC-M affords quantitative removal of the thiocarbonylthio group at the lowest temperatures during solvent-free thermolysis (170 °C).

## Conclusions

Well-defined SMAnh (*M*_*n*_ ≅ 3500 g/mol, *Đ* < 1.2), with either an MAnh- or STY-functional ω-chain end, was synthesized using three different CTAs with distinct reactivity. This afforded a library of macro-CTAs with varying ω-chain end chemical composition, which subsequently underwent desulfurization via radical-induced reduction or thermolysis, yielding SMAnh with a hydrogenated or alkene-functionalized chain end, respectively. The effect of ω-chain end chemical composition and end-group removal conditions (solvent type and reaction temperature) on the rate and extent of thiocarbonylthio group removal was investigated. Overall, dithiocarbamate chain ends exhibited the highest lability, and additionally, MAnh-functional chain ends resulted in higher efficiency of desulfurization compared to STY-functional chain ends. Generally, the use of a polar solvent (DMF) and higher temperatures resulted in faster and quantitative thermolysis, but could result in the production of DMF-derived degradation byproducts, which led to partial modification of the MAnh repeat units along the SMAnh backbone. However, solvent-free and quantitative desulfurization could be achieved at elevated temperatures (>170 °C), affording the opportunity to successfully thermolyze SMAnh chain ends using an inexpensive and “green” protocol without compromising the integrity of the repeat units along the backbone. The work presented here therefore provides an effective and reliable protocol for the desulfurization of SMAnh-type copolymers, for incorporation into synthetic protocols that utilize MAnh-functional copolymers for downstream functionalization. This study further suggests that the presence of an electron-deficient monomer adjacent to the thiocarbonylthio moiety of a RAFT agent leads to increased lability and therefore more efficient end-group removal.

## Supplementary Material



## Notes

All data relevant to the manuscript have been compiled in the ESI. This includes additional experimental data, UV–vis spectra, NMR spectra, ATR-FTIR spectra, and SEC traces (PDF)

## †

Leibniz-Institut für Polymerforschung Dresden e.V., Hohe Straße 6, D-01069 Dresden, Germany

## Abbreviations

CRP: Controlled Radical Polymerization
RAFT: Reversible addition–fragmentation chain transfer
CTAs: chain transfer agents
SMAnh: poly­(styrene-*alt*-maleic anhydride)
STY: styrene
MAnh: maleic anhydride
TTC: trithiocarbonate
DTB: dithiobenzoate
DTC: dithiocarbamate
