# Supplementary material for: Exploring the Removal of Thiocarbonylthio Chain Ends from Poly(styrene-alt-maleic anhydride) Copolymers
Source: Macromolecules. 2025 Sep 3;58(18):10084–93. doi: 10.1021/acs.macromol.5c01887 (PMC12462245; doi:10.1021/acs.macromol.5c01887)
Supplement: Supplementary file 1 [file ma5c01887_si_001.pdf]

Exploring the removal of thiocarbonylthio chain ends from poly(styrene-*alt*-maleic anhydride) copolymers

Michael-Phillip Smith, ‡ Lauren E. Ball,†‡ Ilanie Wessels, Bert Klumperman*

Department of Chemistry and Polymer Science, Stellenbosch University, Matieland 7602, South Africa.

†Leibniz-Institut für Polymerforschung Dresden e.V., Hohe Straße 6, D-01069 Dresden, Germany.

‡Both authors contributed equally to this work.

Table of Contents

Equipment and analysis preparation 1

UV-Vis spectroscopy 1

Size exclusion chromatography 1

Nuclear magnetic resonance spectroscopy 1

Attenuated Total Reflectance Fourier Transform Infrared (ATR-FTIR) Spectroscopy 1

Radical-induced reduction 2

Solvated CTA thermolysis 8

Computational data 16

Computational methods 16

Atom coordinates 17

References 26

Equipment and analysis preparation

UV-Vis spectroscopy

Analysis was conducted on a Shimadzu UV-1800 Spectrophotometer with range limits 190 – 1100 nm and a double beam (Deuterium lamp and Tungsten-Halogen lamp), with a wavelength accuracy ± 0.1 nm, wavelength reproducibility ± 0.1 nm, absorbance range of -4 – 4 and a silicon photodiode detector. Samples were analyzed within a range of 215 – 500 nm and at varying concentrations in 1,4-dioxane based on the CTA attached to the copolymer (TTC = 0.2 $mg \cdot mL^{-1}$; DTC = 0.1 $mg \cdot mL^{-1}$; DTB = 0.1 $mg \cdot mL^{-1}$).

Size exclusion chromatography

The system is composed of an Agilent 1260 HPLC instrument, a quaternary pump, a column compartment thermostated at 30 °C, a differential refractometer (30 °C), and a diode array UV detector (254 nm and 320 nm). The system contains two Agilent Technologies PLgel 5 Mixed-C columns (300 × 7.5 mm inner diameter) and a PLgel 5 Guard column (50 × 7.5 mm i.d.). The system runs on a THF (5% v/v AcOH with 0.125% BHT) solvent system with an analysis flow rate of 1.0 $mL \cdot min^{-1}$, with an injection volume of 100 µL. The system was calibrated using polystyrene calibration standards of a molar mass range of 580 – 2.0 × 106 $g \cdot mol^{-1}$. Samples were pre-dissolved at 2 $mg \cdot mL^{-1}$ for 24 hours prior to analysis. Samples were thereafter filtered through a RC syringe filter (Sartorius, pore size = 0.45 µm) to remove any remaining particulate.

Nuclear magnetic resonance spectroscopy

Analysis was conducted on a Bruker Ascend (400 or 600 MHz) spectrometer. Samples were dissolved in $(CD_3)_2CO$ at 0.1 $g \cdot mL^{-1}$.

Attenuated Total Reflectance Fourier Transform Infrared (ATR-FTIR) Spectroscopy

Analysis was completed on a Thermo Scientific Nicolet iS10 Smart iTR spectrometer utilizing 128 scans over the wavelength range of 700–4000 cm^{-1}, with a background spectrum (128 scans) acquired prior to each sample.

Equation S1. Monomer conversion calculation for copolymerizations in Table 2 (main text).

$$\alpha = \left(1 - \frac{I_{tx}^{Monomer}}{I_{t0}^{Monomer}}\right) \times 100$$

Equation S2. M1/M2 refers to S and MAnh comonomers.

$$M_n^{theo} = \frac{[M1] \times MW_{M1} \times \alpha_{M1}}{[CTA]} + \frac{[M2] \times MW_{M2} \times \alpha_{M2}}{[CTA]} + MW_{CTA}$$

Radical-induced reduction

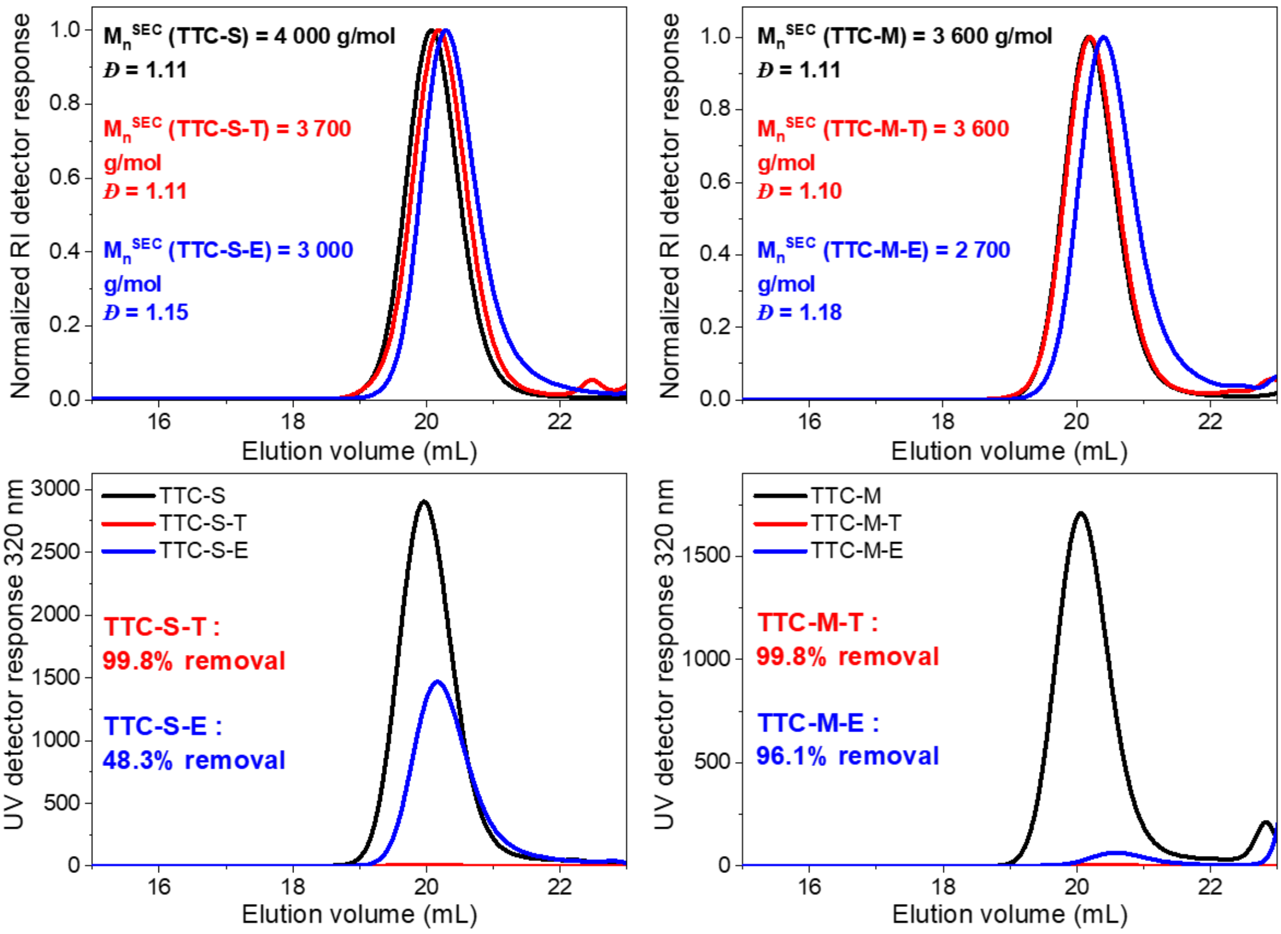

Figure S1. SEC analysis of SMAnh copolymers before (TTC-S or TTC-M) and after radical induced reduction, using TTMSS (indicated by 'T' in the sample name) or EPHP (indicated by 'E' in the sample name). Analysis conducted using THF (5% AcOH) as the mobile phase and PS calibration standards.

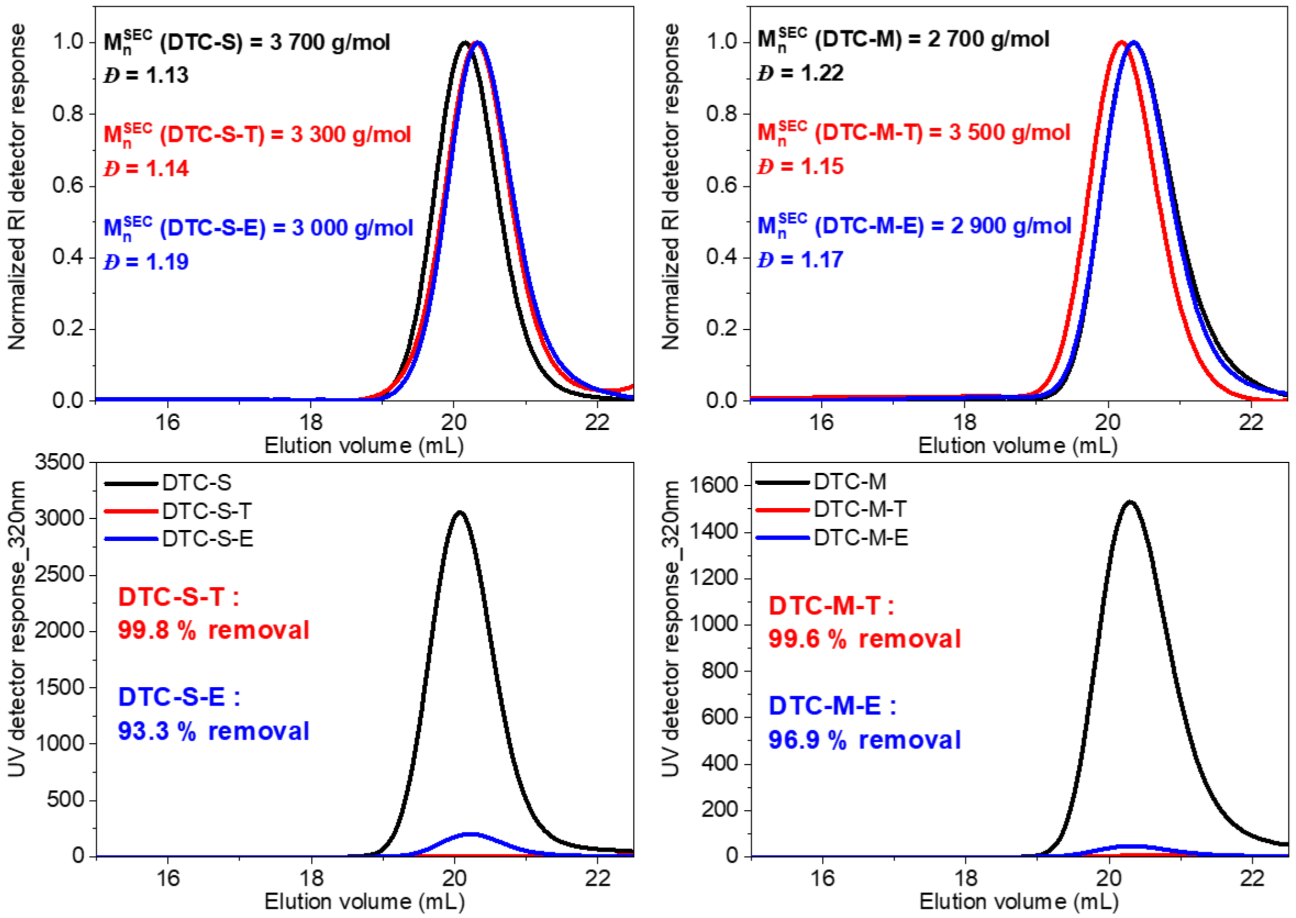

Figure S2. SEC analysis of SMAnh copolymers before (DTC-S or DTC-M) and after radical induced reduction, using TTMSS (indicated by 'T' in the sample name) or EPHP (indicated by 'E' in the sample name). Analysis conducted using THF (5% AcOH) as the mobile phase and PS calibration standards.

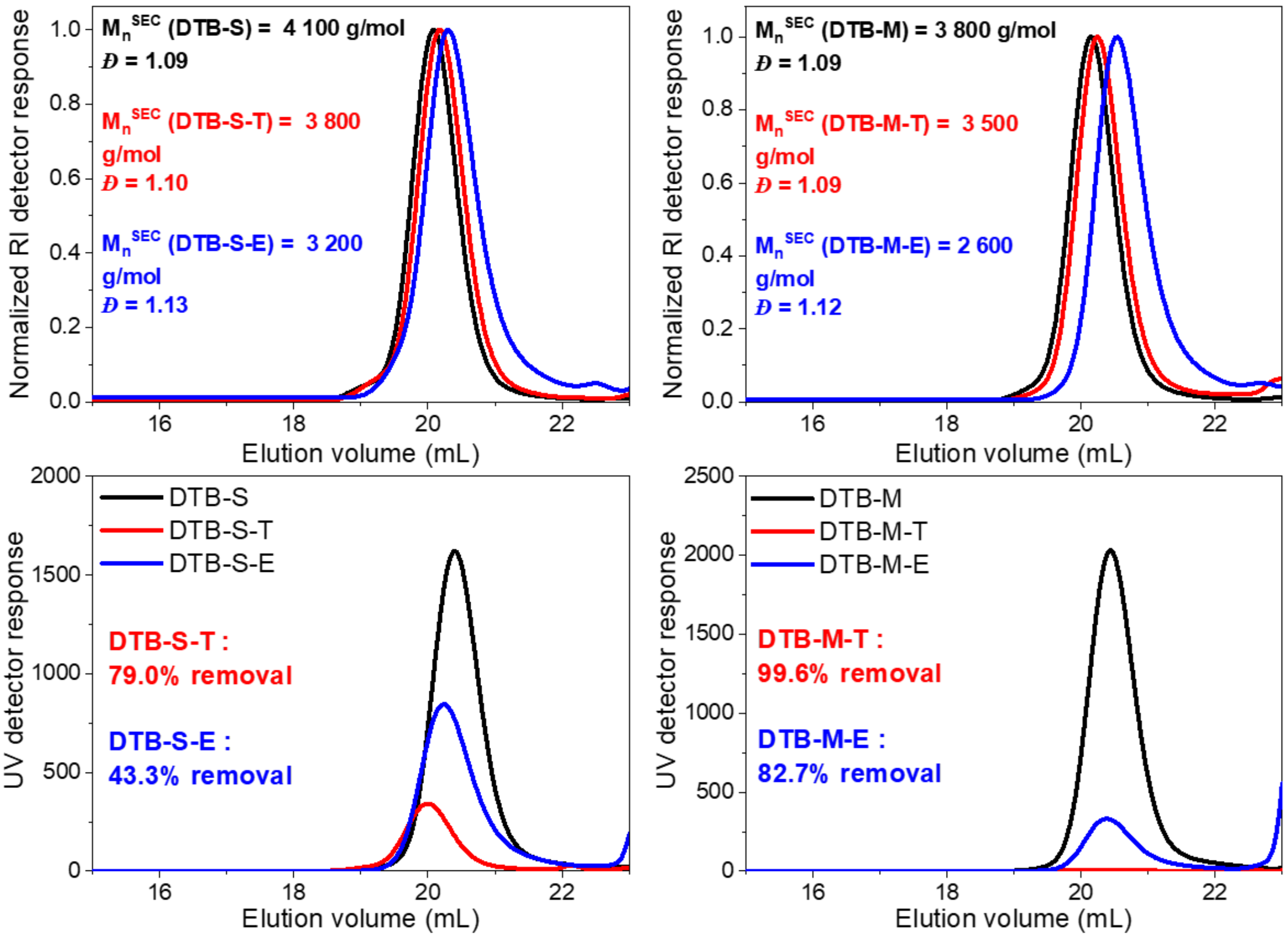

Figure S3. SEC analysis of SMAnh copolymers before (DTB-S or DTB-M) and after radical induced reduction, using TTMSS (indicated by 'T' in the sample name) or EPHP (indicated by 'E' in the sample name). Analysis conducted using THF (5% AcOH) as the mobile phase and PS calibration standards.

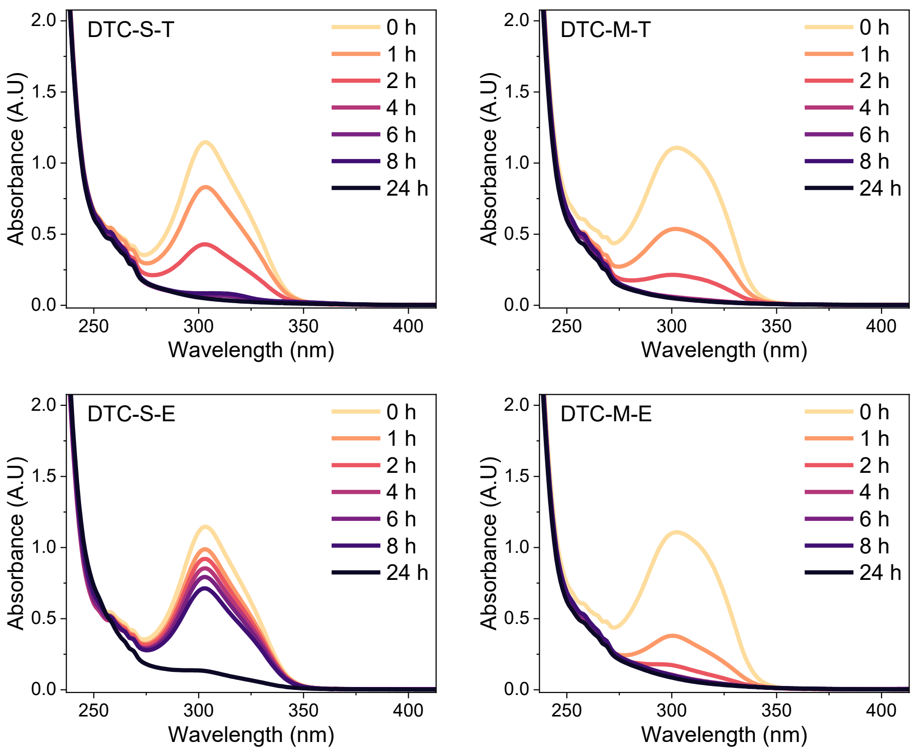

Figure S4. UV-Vis spectroscopic analysis of DTC-SMAnh copolymers in 1,4-dioxane, undergoing TTMSS (T) or EPHP (E) mediated radical induced reduction.

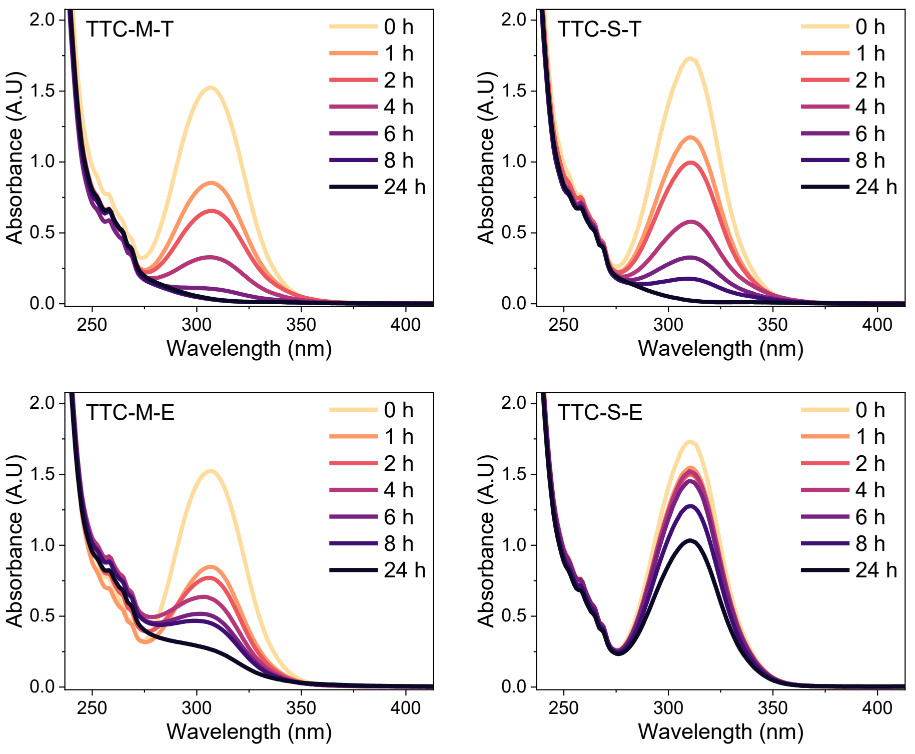

Figure S5. UV-Vis spectroscopic analysis of TTC-SMAnh copolymers in 1,4-dioxane, undergoing TTMSS (T) or EPHP (E) mediated radical induced reduction.

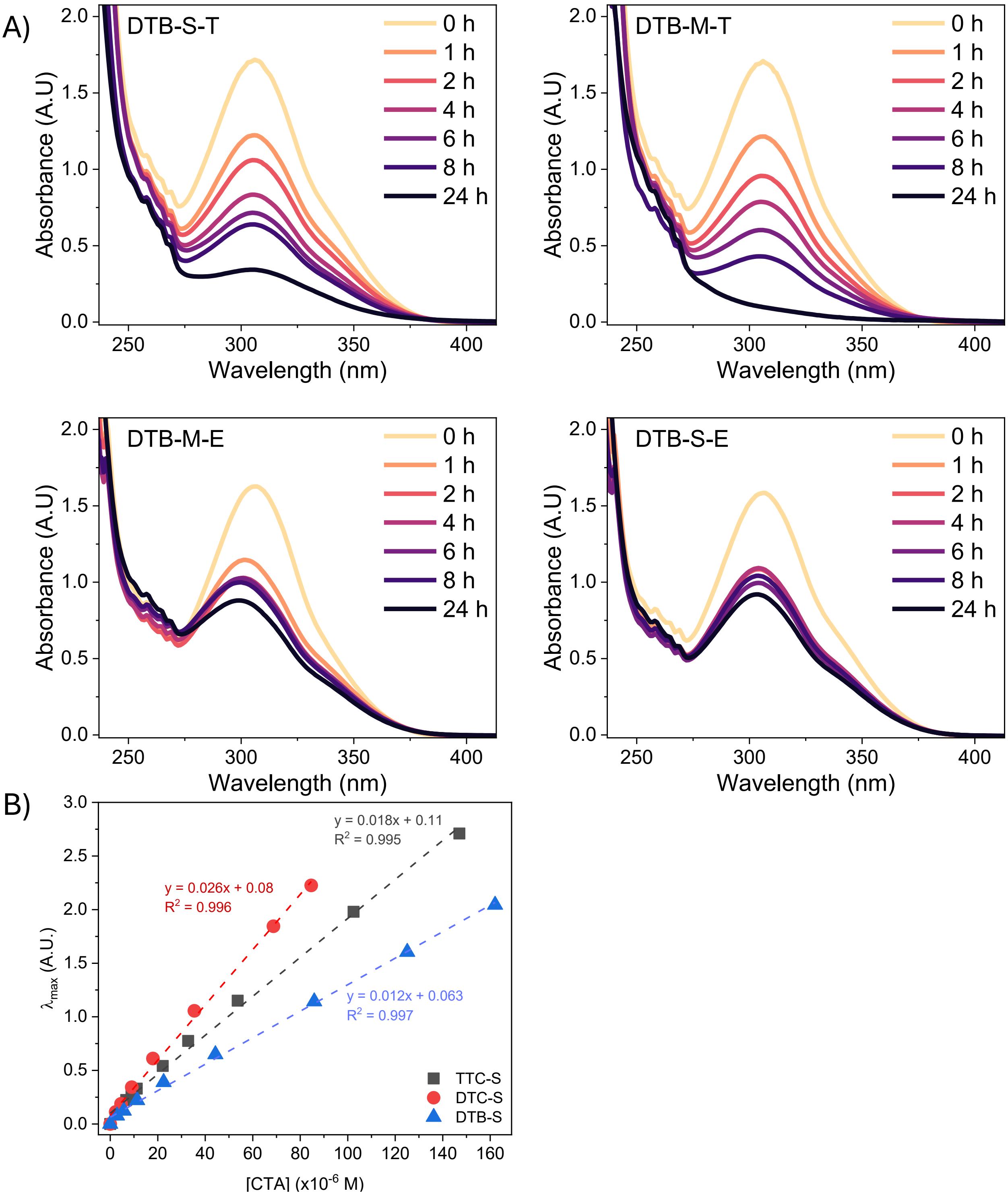

Figure S6. A) UV-Vis spectroscopic analysis of DTB-SMAnh copolymers in 1,4-dioxane, undergoing TTMSS (T) or EPHP (E) mediated radical induced reduction. B) Representative calibration curves generated for SMAnh copolymers with a S-functional chain end. Similar curves were generated for copolymers with MAnh-functional chain ends.

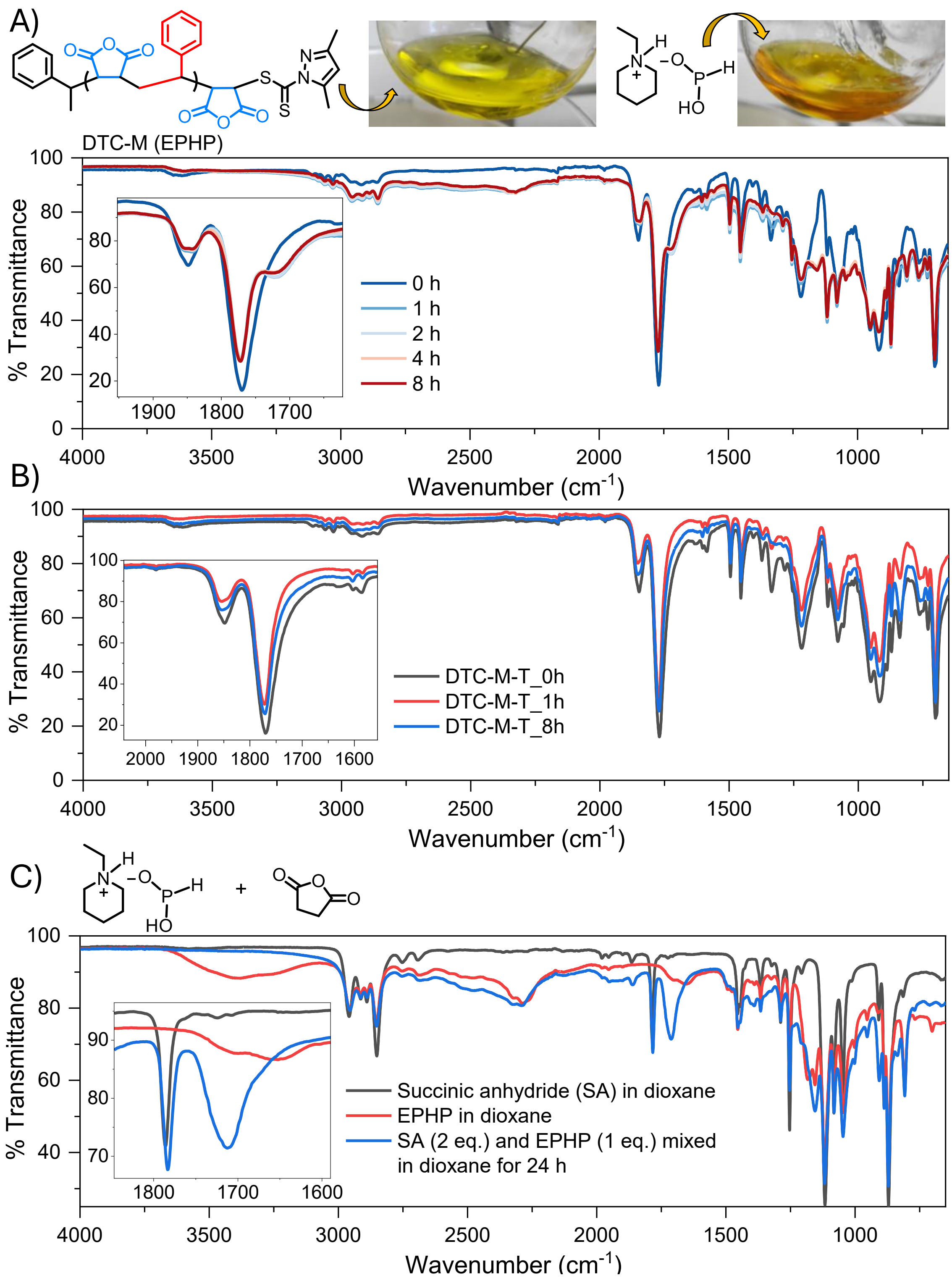

Figure S7. ATR-FTIR spectroscopic analysis of DTC-M before and after addition of EPHP (A) or TTMSS (B). The images indicate the colour of the solution before addition (yellow) and after addition (orange) of EPHP. To assess whether the interaction between EPHP and MAnh repeat units is plausible, EPHP and succinic anhydride were mixed for 24 h in dioxane and the mixture analysed via ATR-FTIR spectroscopy. The signal corresponding

to the O-H stretch of EPHP at 3050-3660 cm^{-1} disappears when mixed with succinic anhydride and additionally, a new signal appears at 1712 cm^{-1}, which is absent in the ATR-FTIR spectra of succinic anhydride or EPHP in dioxane. This suggests that EPHP likely interacts with the MAnh repeat units of SMAnh.

Solvated CTA thermolysis

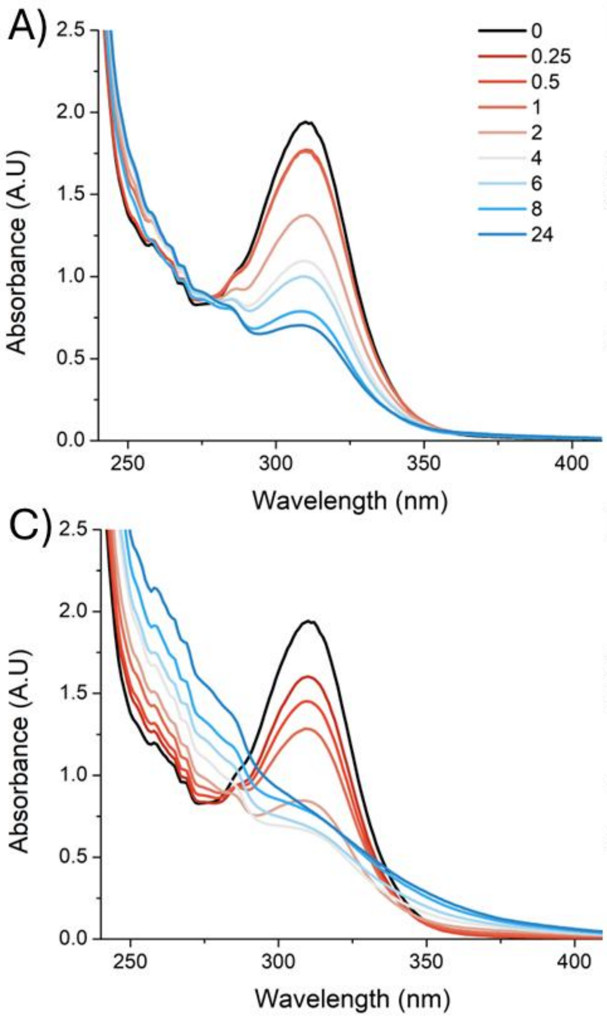

Figure S8. A) UV-Vis spectroscopic analysis of TTC-S copolymers in A) DMF at 100 °C; B) DMF at 130 °C; C) DMF at 160 °C; and D) dioxane at 100 °C; over time (h) to determine the rate of thermolytic cleavage of the thiocarbonylthio group. Similar curves were generated for other copolymers with styrene and MAnh-functional chain ends and different thiocarbonylthio groups present i.e. DTC, TTC or DTB.

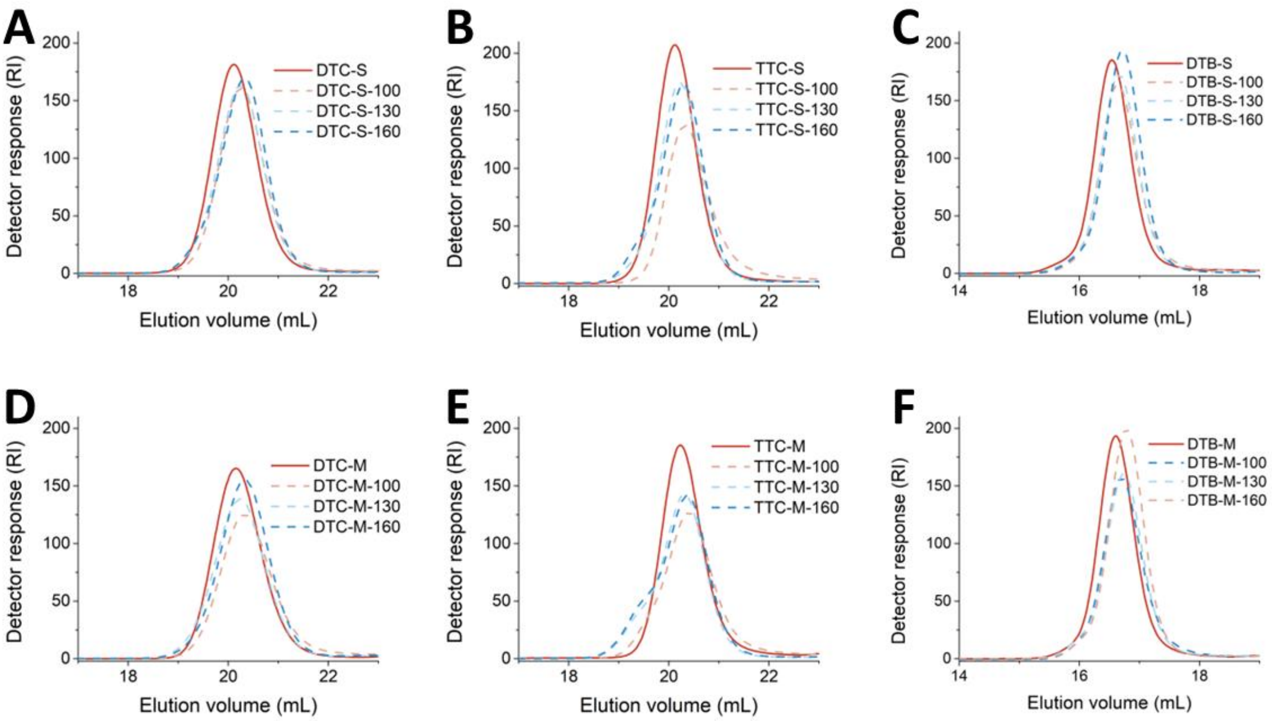

Figure S9. SEC traces of DMF solvated thermolytic cleavage of varying CTAs present on SMAnh copolymers. Samples codes: (CTA Z-group) – (terminal monomer) – (temperature of thermolysis)

Table S1. Summary of SEC data of the solvated thermolysis of SMAnh copolymers with variable terminal monomers and different CTAs in DMF.

Copolymer[a]	M_n^{SEC} ($g{\cdot}mol^{-1}$)[b]	*Đ*[b]	Copolymer[a]	M_n^{SEC} ($g{\cdot}mol^{-1}$)[b]	*Đ*[b]	Copolymer[a]	M_n^{SEC} ($g{\cdot}mol^{-1}$)[b]	*Đ*[b]
TTC-M	3400	1.12	DTC-M	3600	1.14	DTB-M	3600	1.12
TTC-M-100	3000	1.20	DTC-M-100	3100	1.19	DTB-M-100	3000	1.21
TTC-M-130	3600	1.19	DTC-M-130	3500	1.18	DTB-M-130	3200	1.16
TTC-M-160	3500	1.21	DTC-M-160	3300	1.17	DTB-M-160	3100	1.13
TTC-S	3800	1.12	DTC-S	3800	1.13	DTB-S	3800	1.14
TTC-S-100	2900	1.18	DTC-S-100	3400	1.15	DTB-S-100	3200	1.19
TTC-S-130	3700	1.30	DTC-S-130	3500	1.16	DTB-S-130	3400	1.15
TTC-S-160	3600	1.15	DTC-S-160	3400	1.16	DTB-S-160	3300	1.12

a - Copolymer codes are based on **Figure 1** following the naming convention of RAFT CTA-terminal monomer-temperature of thermolysis reaction.
b - Determined via SEC analysis with THF (5% AcOH) as the mobile phase and PS calibration standards.

It is apparent in **Table S1** and **Figure S9** that no chain coupling took place during thermolysis, it is evident that all copolymers had a mass loss equivalent (or close) to the mass of the respective CTA. No copolymer degradation occurred during thermolysis.

NMR spectroscopy was utilized to confirm end group identity, a study by Ball *et al.* assisted with signal assignment.[1] Confirmation of the terminal chain end identity of styrene terminated copolymers is well documented in literature.[2, 3] Via ^{1}H NMR spectroscopy it was possible to discern that the terminal group is the vinyl-trans styrene chain end as has been documented prior.[3]

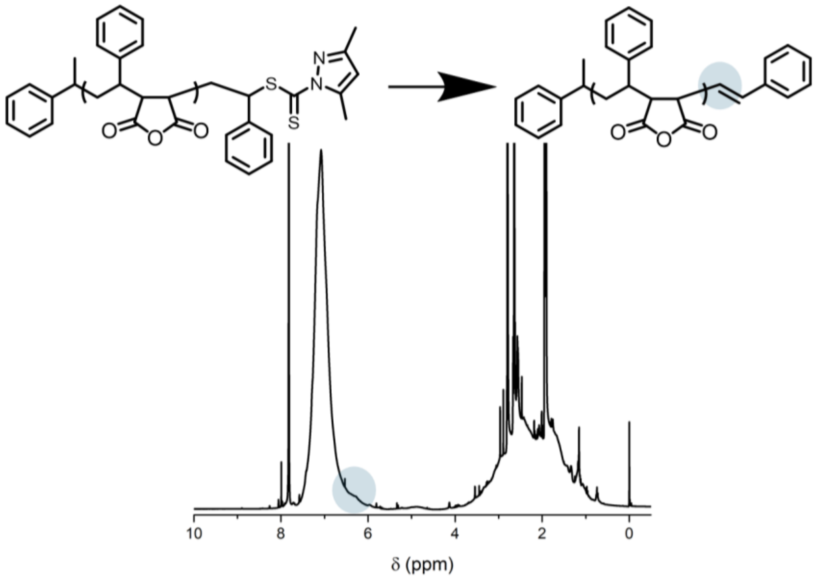

Figure S10. ^{1}H NMR spectrum of DTC-S copolymer after thermolytic removal of the thiocarbonylthio group.

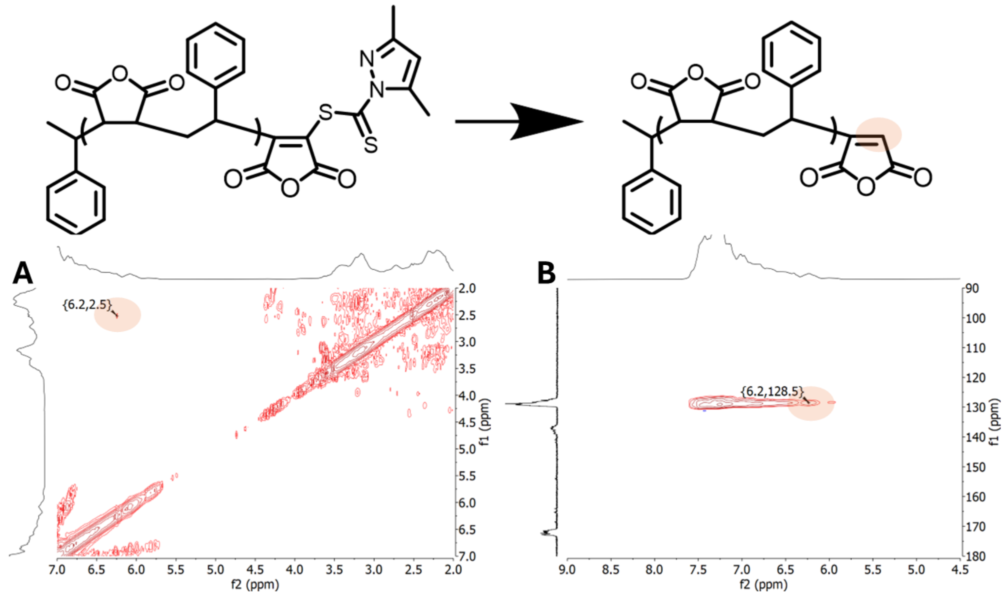

Figure S11. 2D NMR spectra of maleic anhydride terminated copolymer (DTC-M) after thermolysis. A) COSY spectra and B) HSQC spectra.

Signal assignment of maleic anhydride terminated polymers has on the contrary not been characterized. COSY and HSQC NMR spectroscopy was utilized to tentatively assign the terminal group of maleic anhydride terminated copolymer as the vinyl maleic anhydride. This follows the expected trend of the formation of a vinyl chain end during thermolytic cleavage of the thiocarbonylthio group.[2, 3]

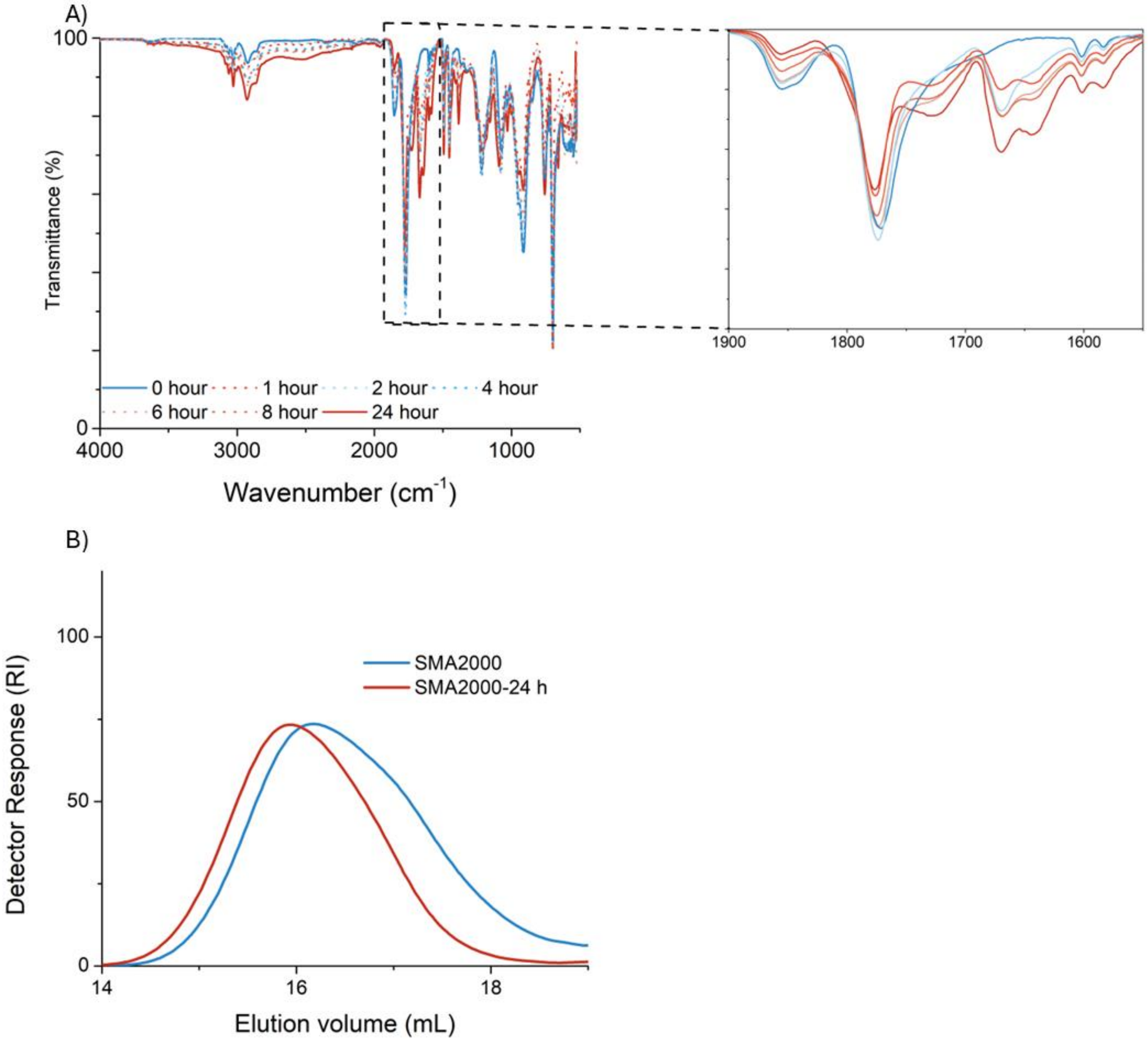

Figure S12. SMA2000 (commercial conventional radical SMAnh 2:1) heated in DMF for 24 hours at 130 °C. A) ATR-FTIR spectra of copolymer over time (h). B) SEC elusion profile, before and after heating of copolymer in DMF for 24 hours at 130 °C.

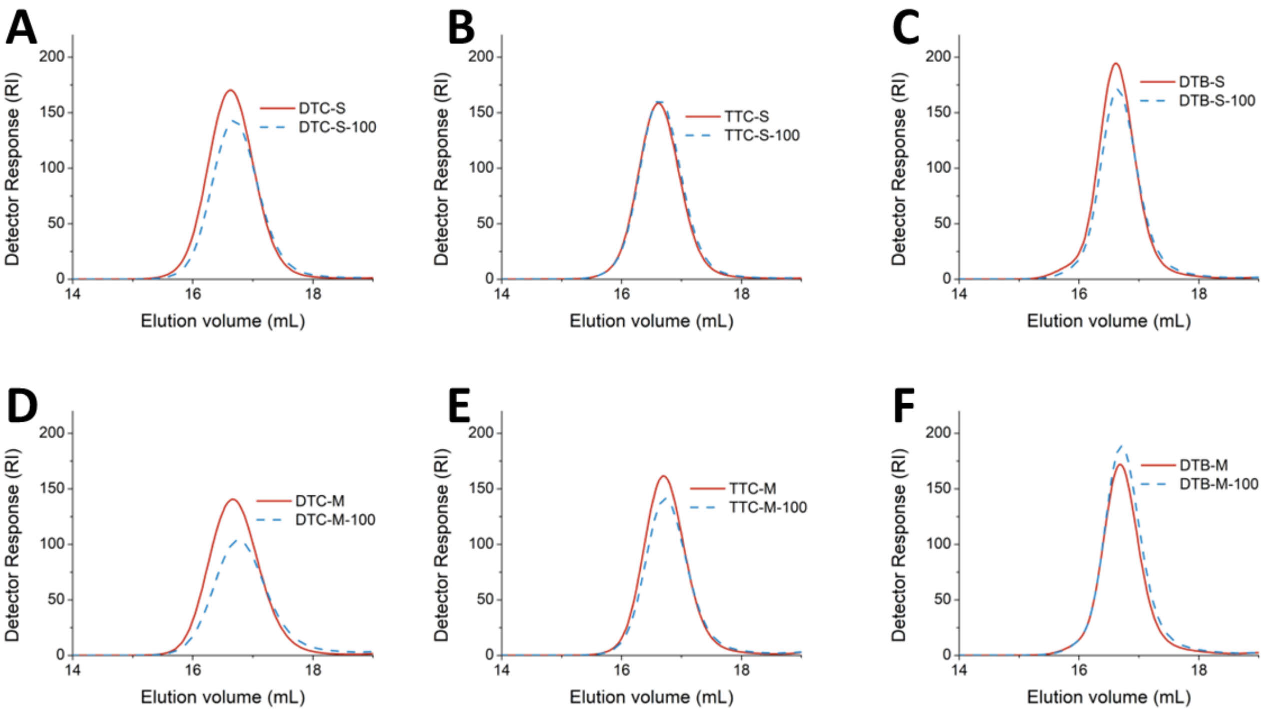

Figure S13. SEC traces of dioxane solvated thermolytic cleavage of varying CTAs present on SMAnh copolymers. Samples codes: (CTA Z-group) – (terminal monomer) – (temperature of thermolysis).

Table S2. Summary of SEC data of the solvated thermolysis of SMAnh copolymers with variable terminal monomers and CTAs in dioxane.

Copolymer [a]	$M_n^{SEC\ b}$ ($g{\cdot}mol^{-1}$)	*Đ* [b]	Copolymer [a]	$M_n^{SEC\ b}$ ($g{\cdot}mol^{-1}$)	*Đ* [b]	Copolymer [a]	$M_n^{SEC\ b}$ ($g{\cdot}mol^{-1}$)	*Đ* [b]
TTC-M	3300	1.16	DTC-M	3400	1.18	DTB-M	3300	1.16
TTC-M-100	3100	1.18	DTC-M-100	2800	1.29	DTB-M-100	3200	1.17
TTC-S	3600	1.15	DTC-S	3700	1.16	DTB-S	3700	1.15
TTC-S-100	3500	1.17	DTC-S-100	3300	1.19	DTB-S-100	3400	1.16

a - Copolymer codes are based on **Figure 1** following the naming convention of CTA – Terminal monomer – temperature of thermolysis reaction.
b - Determined via SEC analysis with THF (5% AcOH) as the mobile phase and PS calibration standards.

It is apparent in **Table S2** and **Figure S13** that no chain coupling took place during thermolysis in dioxane, it is evident that all copolymers had a mass loss equivalent (or close) to the mass of the respective CTA.

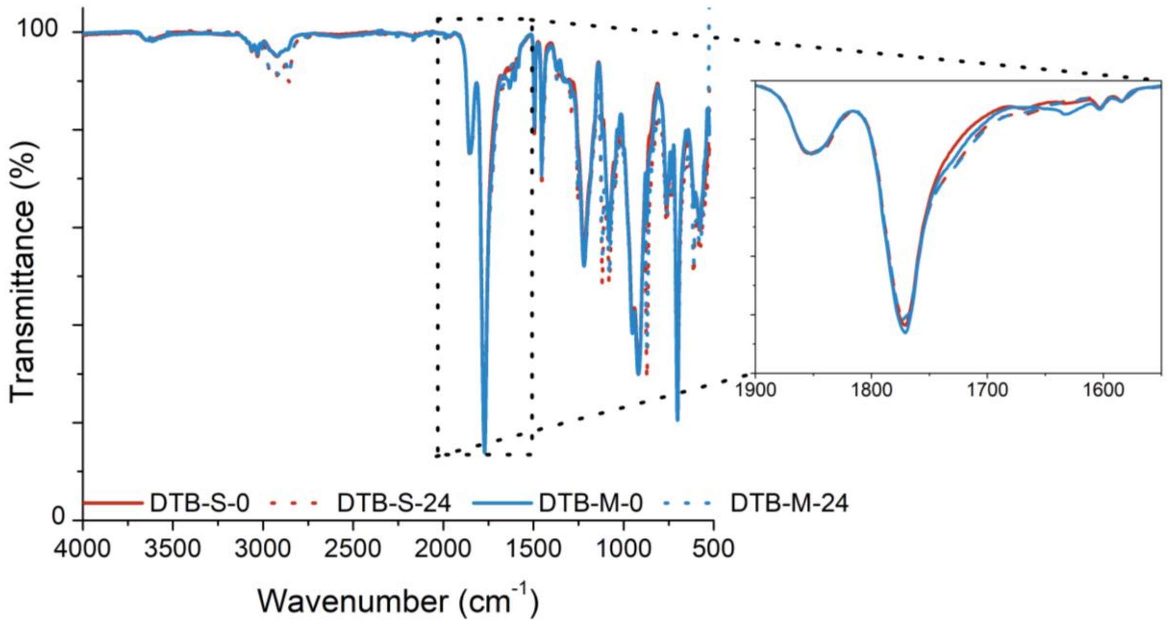

Figure S14. Thermolytic cleavage of DTB-S and M copolymers in dioxane for 24 hours at 100 ℃. No main chain maleic anhydride degradation occurred during thermolytic cleavage.

Solvent-free thermolysis

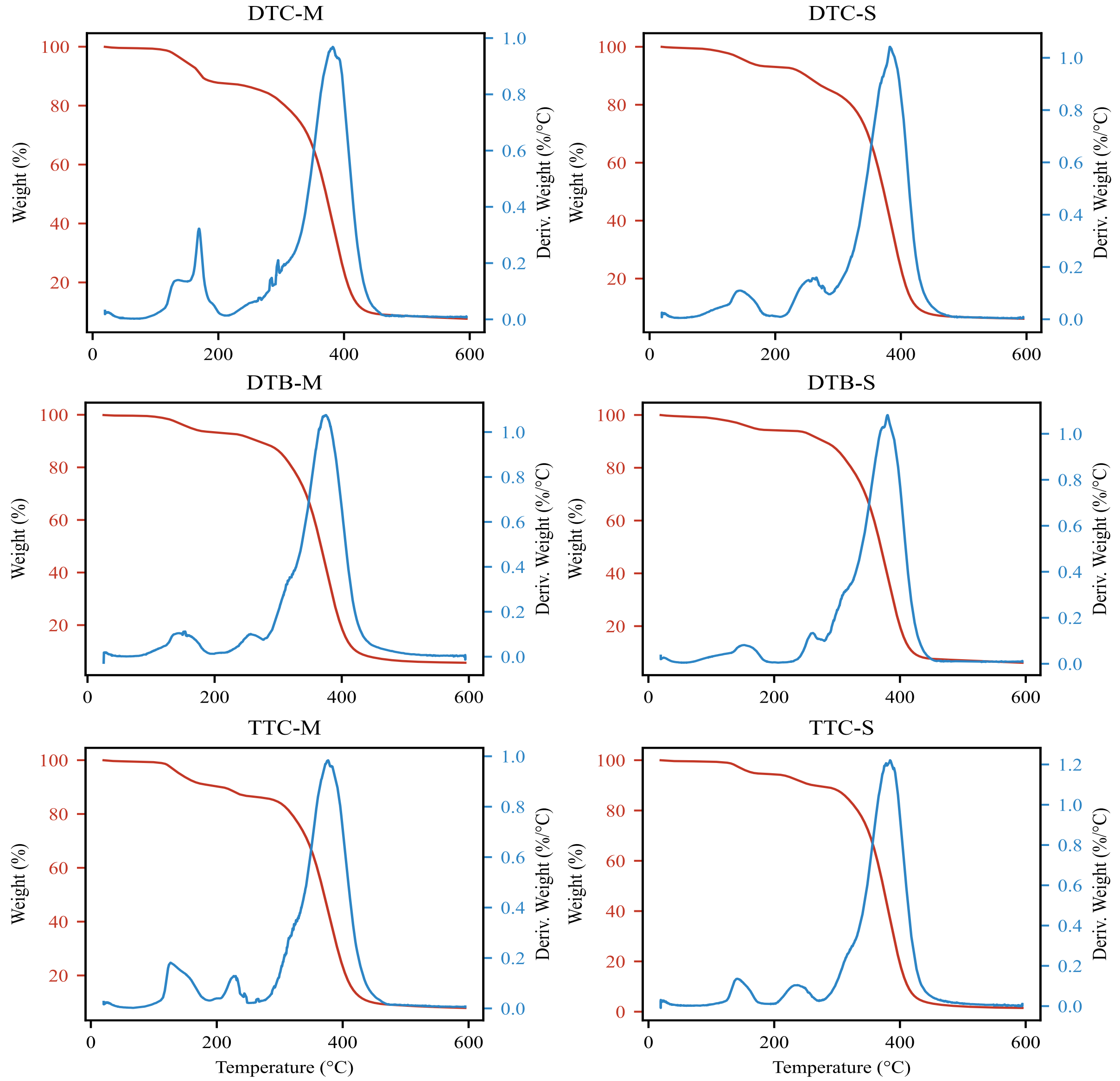

Figure S15. Weight percent and derivative weight percent of the DTC, DTB and TTC polymers as a function of temperature following a ramp to 600 °C.

Table S3. Summary of SEC data of the solvent free thermolysis (2.5 hours) of SMAnh copolymers with variable terminal monomers and CTAs.

Copolymer	M_n^{SEC} (g·mol^{-1})	*Đ*	Copolymer[a]	M_n^{SEC} (g·mol^{-1})	*Đ*	Copolymer[a]	M_n^{SEC} (g·mol^{-1})	*Đ*
TTC-M	3300	1.16	DTC-M	3400	1.18	DTB-M	3300	1.16
TTC-M-230	3000	1.21	DTC-M-170	3000	1.23	DTB-M-260	3200	1.17
TTC-S	3600	1.15	DTC-S	3700	1.16	DTB-S	3700	1.15
TTC-S-235	3600	1.16	DTC-S-255	3300	1.21	DTB-S-260	3500	1.15

a - Copolymer codes are based on **Figure 1** following the naming convention of CTA – Terminal monomer – temperature of thermolysis reaction.
b - Determined *via* SEC analysis with THF (5% AcOH) as the mobile phase and PS calibration standards.

Table S4. Summary of the temperatures required for removal of the thiocarbonylthio groups of different polymers based on TGA data.

Polymer	Thiocarbonylthio group removal temperature (°C)
TTC-S	235
TTC-M	230
DTB-S	260
DTB-M	260
DTC-S	255
DTC-M	170

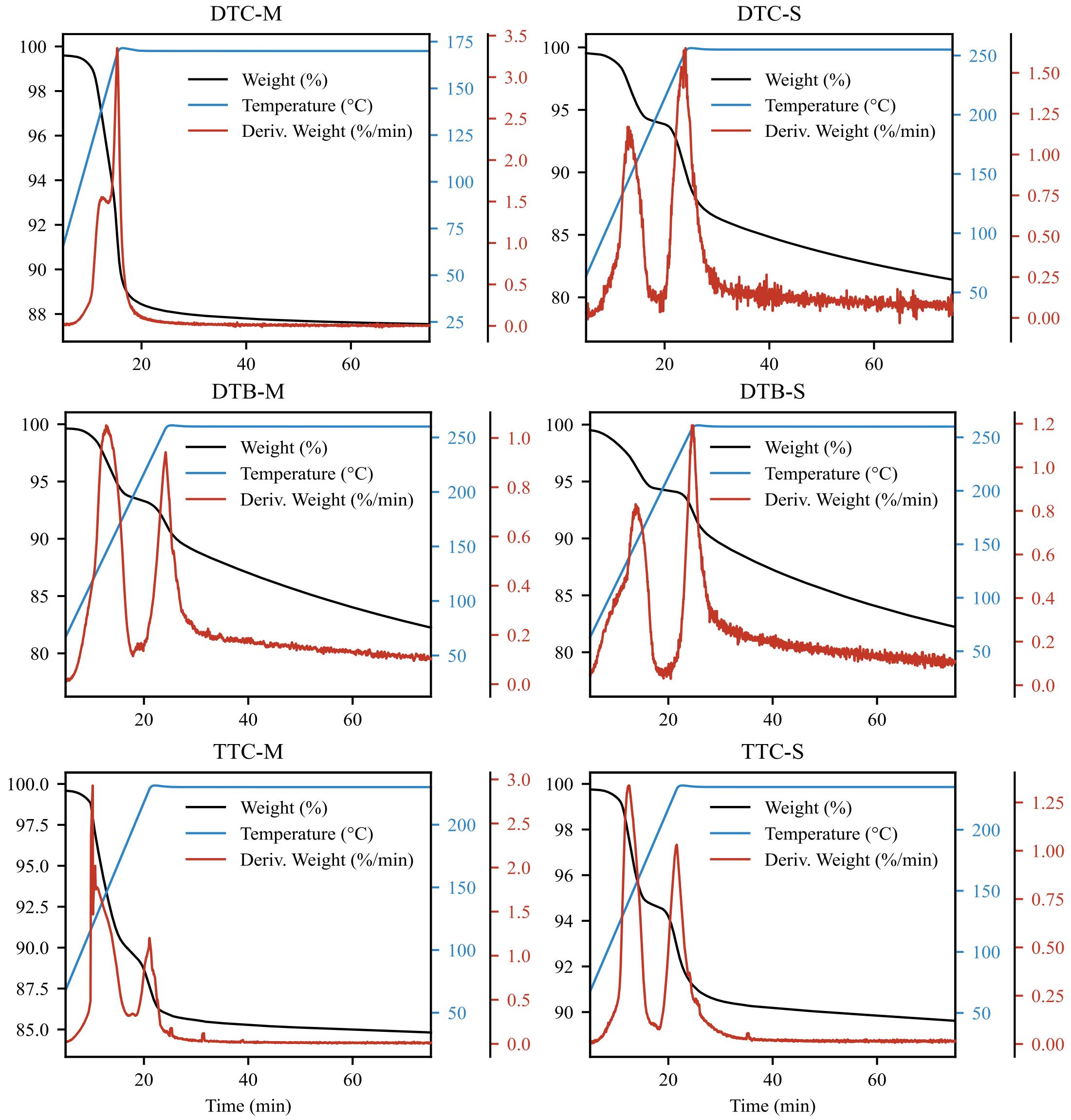

Figure S16. Weight percent and derivative weight change of the DTB and TTC polymers as a function of time following a ramp to the thiocarbonylthio group removal temperature which was held constant for 150 minutes.

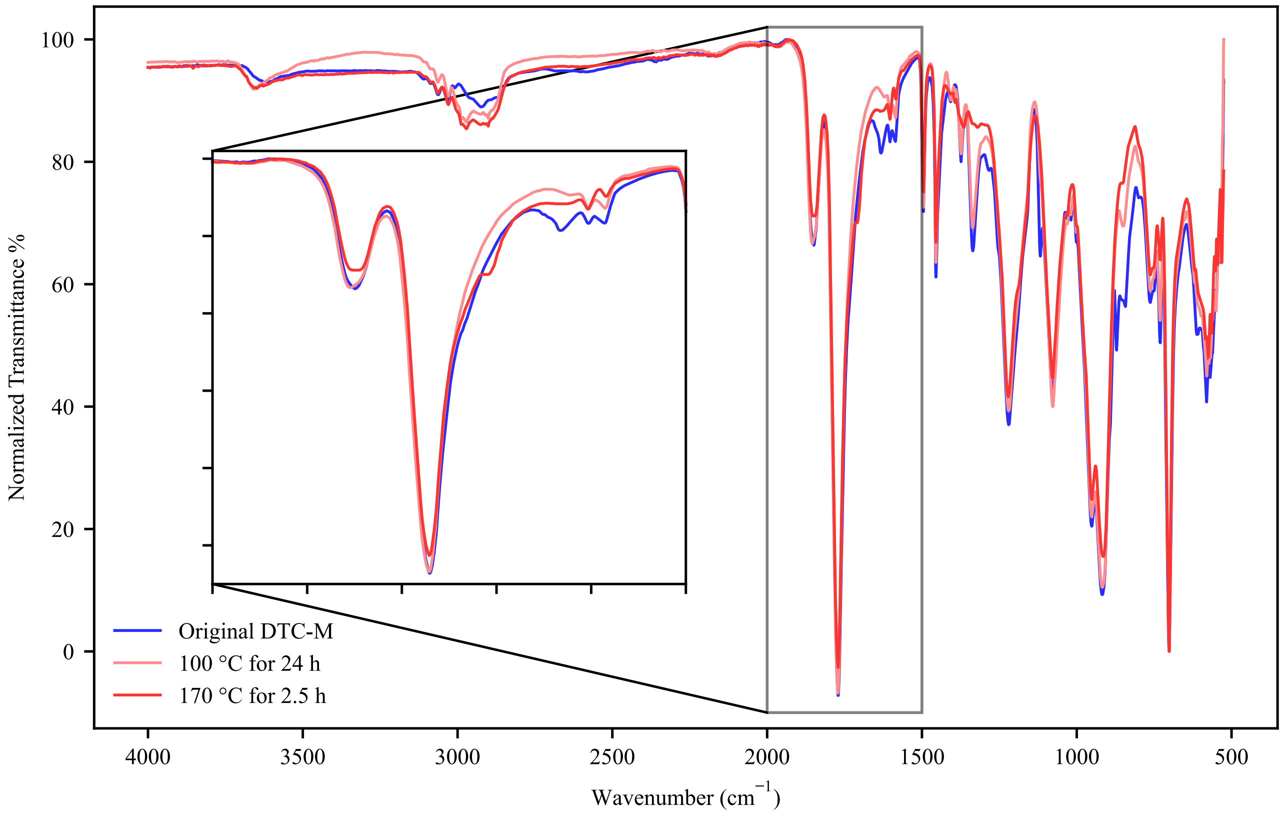

Figure S17. ATR-FTIR tracking of backbone in the solvent-free thermolysis of DTB-M copolymer at variable temperatures and time (h).

Computational data

Computational methods

Analysis was conducted on the Jaguar module of the Schrödinger Suite (2021-4),[4] *via* the Maestro graphical interface.[5] Molecular geometries were optimized using density functional theory with M06,[6-9] using the 6-31+G** basis set.[10-14] Jaguar's (*via* the Maestro interface) check_min=1 function was utilized to confirm geometries corresponded to minima. All calculations were treated with unrestricted spin. A THF solvent environment was modelled utilizing the conductor-like polarizable continuum model (CPCM) and compared to the gas-phase optimized structure.[15-17] Basis sets were obtained *via* the Basis Set Exchange.[18]

Atom coordinates

Alkene-MAnh-S

Final energy = -688.585296 hartree

C	4.20690	-3.12260	0.68520
C	3.48000	-2.72900	1.73880
C	2.13060	-2.35820	1.22000
O	2.12710	-2.53360	-0.14650
C	3.37980	-3.00070	-0.52520
O	1.16100	-1.98250	1.81510
O	3.63320	-3.23030	-1.67380
C	3.81080	-2.60520	3.18590
C	5.10420	-3.32580	3.55010
C	3.85100	-1.13550	3.57490
C	3.04810	-0.65660	4.61010
C	3.10840	0.68210	4.99060
C	3.97160	1.55660	4.33520
C	4.77420	1.08620	3.29780
C	4.71290	-0.25190	2.91950
H	5.23030	-3.47570	0.64820
H	2.97640	-3.06270	3.73990
H	5.96350	-2.88880	3.02790
H	5.28820	-3.23820	4.62570
H	5.04920	-4.38930	3.29360
H	2.36590	-1.33750	5.11780
H	2.47580	1.04280	5.79830
H	4.01670	2.60250	4.62920
H	5.44770	1.76390	2.77920
H	5.34210	-0.60940	2.10470

H-MAnh-S

Final energy = -689.811204 hartree

C	4.70320	-3.60540	0.52780
C	4.25420	-2.36640	1.29560
C	2.81020	-2.21550	0.87630
O	2.57180	-3.00170	-0.23880
C	3.66500	-3.78840	-0.53480
O	1.93350	-1.54220	1.33390
O	3.66780	-4.49290	-1.50330
C	4.47360	-2.41490	2.81470
C	5.94540	-2.69590	3.12180
C	4.01810	-1.15650	3.51850
C	3.08470	-1.23050	4.55340
C	2.68020	-0.08500	5.23570
C	3.20670	1.15630	4.88880
C	4.14270	1.24150	3.85960
C	4.54710	0.09390	3.18320
H	5.69940	-3.54450	0.08380
H	4.67730	-4.50570	1.15880
H	4.76870	-1.48220	0.88600
H	3.87380	-3.25380	3.20360
H	6.10820	-2.70630	4.20480
H	6.27000	-3.66450	2.72510
H	6.59150	-1.91960	2.69210
H	2.66180	-2.19850	4.82130
H	1.94830	-0.16370	6.03640
H	2.89030	2.05280	5.41650
H	4.56100	2.20620	3.58210
H	5.28390	0.18160	2.38480

Alkene-S-MAnh (cis)

Final energy = -688.581399 hartree

C	1.67560	-0.40770	0.45870
C	2.41280	-1.28870	-0.23060

C	1.96010	-2.13500	-1.37900
C	2.53980	-1.78800	-2.75150
C	2.83730	-3.11500	-3.38090
O	2.75780	-4.10420	-2.41800
C	2.35620	-3.58460	-1.20500
O	2.33790	-4.26480	-0.22020
O	3.12810	-3.38510	-4.51010
C	0.27110	-0.02690	0.25200
C	-0.30330	0.12580	-1.01760
C	-1.64120	0.48560	-1.15430
C	-2.42750	0.70550	-0.02580
C	-1.86270	0.58060	1.24280
C	-0.52500	0.22810	1.37860
H	2.16000	0.07130	1.31140
H	3.44880	-1.43960	0.07610
H	0.86020	-2.14800	-1.43100
H	1.88030	-1.20130	-3.39660
H	3.48800	-1.24020	-2.66590
H	0.30830	0.00550	-1.90930
H	-2.06660	0.60610	-2.14780
H	-3.47200	0.98740	-0.13380
H	-2.46590	0.76400	2.12890
H	-0.08420	0.13690	2.37030

Alkene-S-MAnh (trans)

Final energy = -688.585073 hartree

C	1.91890	-0.32770	0.54160
C	2.46100	0.51720	1.60860
C	2.60300	-1.14570	-0.26680
C	1.59610	1.40460	2.26370
C	2.05800	2.23930	3.27710
C	3.39700	2.19840	3.65650
C	4.26810	1.31480	3.01680
C	3.80670	0.48220	2.00520
C	1.96020	-1.95750	-1.34370
C	2.53210	-1.77390	-2.74900
C	2.60260	-3.15800	-3.31750
O	2.38590	-4.07840	-2.30770
C	2.09550	-3.45030	-1.11510
O	1.96660	-4.07790	-0.10400
O	2.82410	-3.52240	-4.43620
H	0.83730	-0.25580	0.40060
H	3.68760	-1.24690	-0.18340
H	0.54810	1.43840	1.96850
H	1.37010	2.92170	3.77070
H	3.76210	2.84760	4.44860
H	5.31400	1.27290	3.31230
H	4.50070	-0.20580	1.52590
H	0.87590	-1.76720	-1.34900
H	1.94260	-1.12490	-3.40040
H	3.55440	-1.37340	-2.72500

H-S-MAnh

Final energy = -689.812721 hartree

C	2.02370	0.10220	0.06440
C	2.51090	0.76670	1.32090
C	2.24920	-1.40790	0.10540
C	1.68260	0.86130	2.44300
C	2.14640	1.43470	3.62450
C	3.45020	1.92150	3.70020
C	4.28540	1.82980	2.58850
C	3.81690	1.25570	1.40880
C	1.75940	-2.09380	-1.16300
C	2.51950	-1.76860	-2.44430

C	2.69710	-3.09010	-3.12850
O	2.33140	-4.10570	-2.26510
C	1.84770	-3.59560	-1.07640
O	1.55360	-4.32590	-0.17310
O	3.10280	-3.34190	-4.22700
H	0.95080	0.29850	-0.07500
H	2.54240	0.53120	-0.80520
H	3.32070	-1.61950	0.24620
H	1.72370	-1.83570	0.96950
H	0.66230	0.48250	2.38560
H	1.48800	1.50470	4.48740
H	3.81320	2.37230	4.62120
H	5.30380	2.20950	2.63870
H	4.47150	1.18670	0.53950
H	0.68830	-1.87330	-1.29460
H	2.02150	-1.06440	-3.11530
H	3.52250	-1.36880	-2.23750

TTC-H

Final energy = -1390.890115 hartree

C	-0.41690	-1.56070	0.00000
S	-1.84920	-2.60020	0.00000
S	1.13750	-2.11340	0.00000
S	-0.92790	0.11370	0.00000
C	0.64740	1.03000	0.00000
C	0.35350	2.52010	0.00000
C	1.64180	3.33340	0.00000
C	1.37320	4.82940	0.00000
H	-1.15340	-3.75640	0.00000
H	1.21500	0.72930	0.88900
H	1.21500	0.72930	-0.88900
H	-0.24800	2.78530	-0.88250
H	-0.24800	2.78530	0.88250
H	2.24190	3.05890	0.87990
H	2.24190	3.05890	-0.87990
H	2.30350	5.40740	0.00000
H	0.79580	5.12500	-0.88530
H	0.79580	5.12500	0.88530

TTMSS

Final energy = -1517.538003 hartree

Si	2.22120	-0.05640	-0.03300
Si	-0.00260	-0.00240	-0.77760
Si	-1.16210	-1.89820	-0.02690
Si	-1.05940	1.95480	-0.03420
H	-0.00290	-0.00450	-2.27970
C	-0.68130	2.21290	1.79870
C	-0.42880	3.44440	-1.00580
C	-2.93070	1.83790	-0.25260
C	2.25660	-0.52900	1.79510
C	3.20920	-1.33070	-1.01380
C	3.04340	1.63100	-0.23510
C	-1.57630	-1.69250	1.80450
C	-2.76730	-2.10950	-0.99670
C	-0.11850	-3.45690	-0.23710
H	-1.21850	3.09420	2.17360
H	-0.98990	1.34720	2.39890
H	0.39120	2.37610	1.96660
H	-0.92440	4.36130	-0.66000
H	0.65330	3.57270	-0.87840
H	-0.63270	3.32980	-2.07730
H	-3.40550	2.78310	0.04260
H	-3.19750	1.63370	-1.29690
H	-3.35470	1.03820	0.36800

H	3.28720	-0.49760	2.17280
H	1.65280	0.15880	2.40080
H	1.87140	-1.54490	1.95170
H	4.25030	-1.35520	-0.66560
H	2.78690	-2.33640	-0.89670
H	3.21300	-1.08720	-2.08300
H	4.09890	1.57430	0.06260
H	3.00090	1.97220	-1.27670
H	2.55620	2.38990	0.39030
H	-2.06610	-2.59860	2.18500
H	-0.67370	-1.51770	2.40430
H	-2.25870	-0.84810	1.96580
H	-3.30690	-3.00060	-0.64930
H	-3.42530	-1.24100	-0.86910
H	-2.56730	-2.22840	-2.06830
H	-0.69560	-4.34010	0.06800
H	0.18830	-3.59390	-1.28110
H	0.78810	-3.41580	0.38020

TTMSS-radical

Final energy = -1516.900015 hartree

Si	0.14090	-0.43230	0.09080
Si	2.35780	0.31210	0.06280
Si	3.06800	1.17520	-1.99120
Si	2.97150	1.59420	1.91990
C	4.62050	2.45240	1.59750
C	1.65090	2.91000	2.23740
C	3.12960	0.51100	3.45600
C	-0.41330	-0.75500	1.86530
C	-0.97690	0.89520	-0.66130
C	-0.04490	-2.01980	-0.91080
C	2.63700	3.01440	-2.07990
C	4.93360	0.98150	-2.18610
C	2.20360	0.27520	-3.40660
H	4.92780	3.02530	2.48260
H	5.40950	1.72470	1.37110
H	4.54780	3.14950	0.75270
H	1.93600	3.53850	3.09270
H	1.52270	3.55880	1.36090
H	0.68020	2.44950	2.46220
H	3.40790	1.12100	4.32620
H	2.18130	0.00760	3.68130
H	3.89820	-0.25890	3.31760
H	-1.44400	-1.13440	1.87400
H	0.22830	-1.49810	2.35470
H	-0.38720	0.16450	2.46420
H	-2.02630	0.57040	-0.63440
H	-0.89450	1.83880	-0.10640
H	-0.70890	1.09350	-1.70700
H	-1.09110	-2.35420	-0.90280
H	0.25590	-1.86480	-1.95440
H	0.57450	-2.82340	-0.49440
H	2.93920	3.43090	-3.05090
H	1.55780	3.17390	-1.95740
H	3.15360	3.57830	-1.29240
H	5.26250	1.40010	-3.14700
H	5.46910	1.50490	-1.38450
H	5.22440	-0.07550	-2.15530
H	2.56230	0.65660	-4.37210
H	2.40150	-0.80320	-3.37100
H	1.11660	0.42300	-3.36810

TTC-MAnh-S

Final energy = -2079.499840 hartree

S	-1.87560	0.13290	0.81490
C	-0.68480	-1.07900	0.39920
S	0.55100	-1.05520	1.69320
S	-0.69730	-2.04460	-0.93210
C	1.76770	-2.23640	1.06680
C	2.46900	-3.17770	2.05240
C	3.76090	-3.44800	1.31760
O	3.97690	-2.46800	0.36610
C	2.89790	-1.61060	0.29150
O	4.55900	-4.32600	1.47800
O	2.93870	-0.60170	-0.34680
C	-3.07150	0.03220	-0.55990
C	-4.09700	1.14150	-0.40150
C	-5.11840	1.10850	-1.53210
C	-6.13760	2.23070	-1.41480
C	2.71480	-2.63470	3.48910
C	3.36960	-3.69380	4.37210
C	3.45610	-1.31340	3.52710
C	2.79750	-0.15540	3.95190
C	3.45120	1.07450	3.98060
C	4.78230	1.16550	3.58460
C	5.45890	0.01580	3.18240
C	4.80500	-1.21250	3.16360
H	1.21430	-2.83730	0.31990
H	1.93200	-4.12800	2.15150
H	-2.51100	0.13180	-1.49730
H	-3.53760	-0.96070	-0.53920
H	-4.61530	1.04510	0.56430
H	-3.59100	2.11830	-0.39260
H	-4.59150	1.17840	-2.49530
H	-5.62860	0.13350	-1.53050
H	-6.87030	2.19890	-2.22910
H	-6.68780	2.16440	-0.46720
H	-5.64590	3.21170	-1.44520
H	1.70400	-2.45640	3.88480
H	3.41600	-3.33420	5.40510
H	2.79290	-4.62650	4.35810
H	4.39100	-3.92600	4.05180
H	1.75790	-0.21800	4.27200
H	2.91650	1.96080	4.31300
H	5.29320	2.12480	3.59780
H	6.50310	0.07300	2.88510
H	5.36510	-2.09820	2.86540

TTC-S-MAnh

Final energy = -2079.511921 hartree

C	0.87970	3.12010	-0.30820
S	0.93650	1.85610	0.92940
S	2.07570	3.49760	-1.38030
C	2.56700	1.03160	0.64950
C	2.97890	0.41470	1.95980
C	2.46310	0.03950	-0.50460
C	4.09370	0.91290	2.63840
C	4.50030	0.34620	3.84400
C	3.79080	-0.72210	4.38740
C	2.67410	-1.22200	3.71950
C	2.27190	-0.65930	2.51180
S	-0.67870	3.92120	-0.17600
C	-0.56320	5.25690	-1.42120
C	-1.81920	6.11000	-1.37230
C	-3.09440	5.38850	-1.78910
C	-4.28350	6.33340	-1.85040
C	3.76710	-0.70500	-0.80520
C	3.63700	-1.74970	-1.90770

C	4.76960	-1.47090	-2.84830
O	5.42570	-0.32220	-2.45070
C	4.87410	0.19610	-1.29630
O	5.29930	1.21130	-0.82230
O	5.12690	-2.07120	-3.82120
H	3.25940	1.84110	0.38760
H	1.69900	-0.71620	-0.27860
H	2.13010	0.56630	-1.40970
H	4.64890	1.74790	2.21260
H	5.37320	0.74110	4.35860
H	4.10620	-1.16430	5.32920
H	2.11350	-2.05320	4.14010
H	1.39620	-1.05820	2.00010
H	0.33530	5.84080	-1.19320
H	-0.43080	4.78990	-2.40510
H	-1.94330	6.54260	-0.36750
H	-1.64950	6.96060	-2.04970
H	-2.93470	4.91500	-2.77010
H	-3.30980	4.57000	-1.08630
H	-5.20300	5.81220	-2.13750
H	-4.45830	6.80710	-0.87560
H	-4.11060	7.13530	-2.58020
H	4.14500	-1.16380	0.12090
H	3.69660	-2.78470	-1.56010
H	2.70000	-1.65140	-2.47100

AIBN-radical

Final energy = -210.597322 hartree

C	0.02790	0.02210	0.26530
C	1.51630	0.02070	0.21020
C	2.25430	-1.27290	0.23360
C	2.21430	1.22340	0.13400
N	2.80320	2.23960	0.07030
H	-0.39250	1.03080	0.25450
H	-0.38300	-0.53830	-0.58720
H	-0.31750	-0.49340	1.17280
H	3.33850	-1.13910	0.19850
H	1.99840	-1.83780	1.14140
H	1.94980	-1.89760	-0.61880

AIBN-TTC-MAnh-S radical

Final energy = -2290.102403 hartree

S	-3.06140	-0.40720	1.61720
C	-1.63020	-1.39440	1.79060
S	-0.05080	-0.62740	1.62510
S	-1.82010	-3.01620	2.39400
C	0.88990	-1.94930	0.78380
C	1.65310	-3.02300	1.57370
C	2.77100	-3.35620	0.61880
O	2.92580	-2.32730	-0.29640
C	1.92920	-1.38780	-0.15870
O	3.49260	-4.31030	0.58570
O	1.95230	-0.35680	-0.76370
C	-2.68460	0.70570	0.20930
C	-2.60240	-0.02820	-1.11490
C	-2.36870	0.92820	-2.27680
C	-2.37570	0.20940	-3.61640
C	2.20100	-2.61950	2.97090
C	2.62770	-3.85840	3.75120
C	3.27300	-1.54780	2.92330
C	2.96020	-0.22390	3.24800
C	3.93000	0.77560	3.20590
C	5.23680	0.46480	2.84270
C	5.56780	-0.85320	2.53580

C	4.59740	-1.84980	2.58030
C	-2.10990	-2.81270	4.28690
C	-1.39330	-1.59560	4.85250
C	-3.60930	-2.77500	4.55710
C	-1.52330	-4.03330	4.82840
N	-1.05920	-5.01340	5.24860
H	0.16240	-2.45050	0.12700
H	1.03630	-3.91870	1.71120
H	-3.52040	1.41670	0.22090
H	-1.76930	1.26830	0.42710
H	-1.78860	-0.77050	-1.07880
H	-3.53370	-0.59200	-1.27650
H	-3.14600	1.70730	-2.26630
H	-1.40960	1.44770	-2.13040
H	-2.19880	0.89920	-4.44870
H	-1.59800	-0.56430	-3.65170
H	-3.34090	-0.28380	-3.79020
H	1.33240	-2.18570	3.48570
H	2.98910	-3.57000	4.74410
H	1.77760	-4.53910	3.88020
H	3.42770	-4.41300	3.24820
H	1.94370	0.02550	3.54990
H	3.66230	1.79740	3.46420
H	5.99660	1.24190	2.80960
H	6.58950	-1.11050	2.26650
H	4.88580	-2.87430	2.35230
H	-1.58960	-1.53690	5.92940
H	-0.31050	-1.64360	4.70070
H	-1.77680	-0.68140	4.38660
H	-3.79480	-2.73810	5.63760
H	-4.03840	-1.87550	4.10190
H	-4.11330	-3.65240	4.14140

AIBN-TTC-S-MAnh radical

Final energy = -2290.110758 radical

C	-0.46660	1.34790	-0.84710
S	0.23960	0.04670	0.09490
S	-1.38650	1.02990	-2.29430
C	2.06950	0.48210	0.09310
C	2.56990	0.77080	1.47870
C	2.80050	-0.66290	-0.60430
C	3.09100	2.03110	1.78130
C	3.58450	2.30860	3.05460
C	3.56100	1.32760	4.04180
C	3.04100	0.06700	3.74930
C	2.55140	-0.20970	2.47710
S	-0.27490	2.94460	-0.15330
C	-0.65630	4.08800	-1.53610
C	-0.23550	5.50070	-1.16970
C	-1.00210	6.10880	-0.00310
C	-0.62250	7.56240	0.22730
C	4.31520	-0.47840	-0.69030
C	5.06700	-1.67260	-1.26500
C	6.07900	-1.07640	-2.19480
O	5.80180	0.26700	-2.36870
C	4.73830	0.66160	-1.58180
O	4.30280	1.77570	-1.66230
O	6.99970	-1.58730	-2.76500
C	-3.05300	0.37820	-1.66410
C	-3.57690	1.20100	-0.49870
C	-3.97890	0.42960	-2.87570
C	-2.84510	-1.00850	-1.25520
N	-2.68450	-2.11440	-0.93240
H	2.13390	1.39000	-0.51610

H	2.61590	-1.60530	-0.06920
H	2.38840	-0.79230	-1.61470
H	3.11200	2.79860	1.00850
H	3.98940	3.29390	3.27310
H	3.94530	1.54270	5.03590
H	3.01670	-0.70330	4.51640
H	2.14180	-1.19690	2.26230
H	-0.11430	3.72890	-2.41720
H	-1.73250	4.03500	-1.74350
H	0.84500	5.52640	-0.96180
H	-0.38970	6.12390	-2.06340
H	-2.08170	6.02580	-0.20090
H	-0.81430	5.52920	0.91310
H	-1.17250	7.99880	1.06790
H	0.44920	7.65800	0.44360
H	-0.83420	8.16890	-0.66280
H	4.70890	-0.23250	0.30870
H	5.56150	-2.30440	-0.52330
H	4.41130	-2.32140	-1.86180
H	-4.55360	0.81280	-0.18630
H	-2.90010	1.16570	0.36180
H	-3.69750	2.24330	-0.81290
H	-4.94970	-0.00830	-2.61920
H	-4.13140	1.47330	-3.16880
H	-3.56440	-0.12230	-3.72570

TTC-AIBN

Final energy = -1600.932067 hartree

C	1.01710	3.58550	-0.84230
S	1.77030	2.53730	0.37430
S	1.57920	3.93240	-2.34790
S	-0.48080	4.18030	-0.13420
C	-1.15070	5.26280	-1.44120
C	-2.49150	5.81180	-0.98470
C	-3.09010	6.74040	-2.03390
C	-4.44640	7.28040	-1.61000
C	3.33700	1.86150	-0.37090
C	4.40380	2.93350	-0.56450
C	3.79710	0.82010	0.65770
C	3.02620	1.15790	-1.61720
N	2.82570	0.52130	-2.56910
H	-0.42480	6.06470	-1.62660
H	-1.24810	4.66840	-2.35840
H	-3.18800	4.98280	-0.78770
H	-2.37450	6.35750	-0.03640
H	-2.39410	7.57230	-2.21990
H	-3.18190	6.19800	-2.98740
H	-4.86820	7.95210	-2.36570
H	-5.16240	6.46340	-1.45190
H	-4.37020	7.84060	-0.66940
H	5.31730	2.46580	-0.95170
H	4.08350	3.70930	-1.26350
H	4.62850	3.38920	0.40520
H	4.72740	0.36090	0.30580
H	3.99590	1.31200	1.61560
H	3.05010	0.03410	0.80410

Radical-MAnh-S

Final energy = -689.156500 hartree

C	3.83900	-1.83250	0.53270
C	2.96130	-2.33080	1.60940
C	2.55190	-3.69230	1.08800
O	3.15520	-3.92720	-0.12050
C	3.94140	-2.82880	-0.50030

O	1.83550	-4.50620	1.60090
O	4.54770	-2.84530	-1.54340
C	3.61750	-2.42920	3.02010
C	4.84350	-3.33170	2.99500
C	3.87160	-1.03610	3.55100
C	2.82210	-0.34150	4.16180
C	3.00150	0.95290	4.63910
C	4.24080	1.57760	4.50990
C	5.29250	0.89690	3.90340
C	5.10980	-0.40070	3.42780
H	4.34490	-0.87500	0.48420
H	2.05180	-1.71640	1.70810
H	2.85230	-2.89510	3.65760
H	5.31990	-3.35990	3.98030
H	4.56930	-4.36000	2.73130
H	5.59410	-2.99260	2.26940
H	1.85240	-0.82860	4.26840
H	2.17460	1.47270	5.11720
H	4.38590	2.58830	4.88370
H	6.26400	1.37490	3.80030
H	5.94710	-0.91650	2.96120

Radical-S-MAnh

Final energy = -689.166497 hartree

C	1.94340	-0.29800	0.46240
C	2.44720	0.53970	1.48070
C	2.35590	-1.71580	0.26650
C	1.98860	1.88030	1.59200
C	2.46480	2.71910	2.58360
C	3.41410	2.25950	3.50400
C	3.87900	0.94370	3.41520
C	3.41010	0.09420	2.42610
C	1.77560	-2.30360	-1.01290
C	2.24530	-1.66270	-2.31370
C	2.52640	-2.81810	-3.22610
O	2.48810	-3.99490	-2.50010
C	2.13400	-3.75640	-1.18830
O	2.13230	-4.64340	-0.38260
O	2.77460	-2.83420	-4.39740
H	1.18660	0.11720	-0.20690
H	3.45590	-1.80010	0.23060
H	2.04150	-2.34060	1.11850
H	1.24960	2.24030	0.87750
H	2.09820	3.74110	2.64730
H	3.78650	2.92000	4.28300
H	4.61490	0.58090	4.12940
H	3.78170	-0.92730	2.37730
H	0.67620	-2.27500	-0.95360
H	1.52810	-0.98370	-2.78230
H	3.18330	-1.10590	-2.18100

References

(1) Ball, L. E.; Smith, M.-P.; Pfukwa, R.; Klumperman, B. An Exploration of the Universal and Switchable RAFT-Mediated Synthesis of Poly(styrene-alt-maleic acid)-*b*-poly(*N*-vinylpyrrolidone) Block Copolymers. *Macromolecules* **2025**, *58* (2), 1060-1076. DOI: 10.1021/acs.macromol.4c02741.

(2) Postma, A.; Davis, T., P.,; Moad, G.; O'Shea, M. S. Thermolysis of RAFT-Synthesized Polymers. A Convenient Method for Trithiocarbonate Group Elimination. *Macromolecules* **2005**, *38 (13)*, 5371-5374.

(3) Stace, S. J.; Fellows, C. M.; Moad, G.; Keddie, D. J. Effect of the Z- and Macro-R-Group on the Thermal Desulfurization of Polymers Synthesized with Acid/Base "Switchable" Dithiocarbamate RAFT Agents. *Macromol Rapid Commun* **2018**, *39* (19), e1800228. DOI: 10.1002/marc.201800228

(4) Bochevarov, A. D.; Harder, E.; Hughes, T. F.; Greenwood, J. R.; Braden, D. A.; Philipp, D. M.; Rinaldo, D.; Halls, M. D.; Zhang, J.; Friesner, R. A. Jaguar: A high-performance quantum chemistry software program with strengths in life and materials sciences. *International Journal of Quantum Chemistry* **2013**, *113* (18), 2110-2142. DOI: 10.1002/qua.24481.

(5) *Maestro*; Schrödinger. Suite, 2021-4. (computational software).

(6) Vosko, S. H.; Wilk, L.; Nusair, M. Accurate spin-dependent electron liquid correlation energies for local spin density calculations: a critical analysis. *Canadian Journal of Physics* **1980**, *58* (2), 1200-1211.

(7) Stephens, P. J.; Devlin, F. J.; Chabalowski, C. F.; Frisch, M. J. Ab initio calculation of vibrational absorption and circular dichroism spectra using density functional force fields. *The Journal of Physical Chemistry* **1994**, *98* (45), 11623-11627.

(8) Becke, A. D. Density-functional thermochemistry. III. The role of exact exchange. *The Journal of Chemical Physics* **1993**, *98* (7), 5648-5652. DOI: 10.1063/1.464913.

(9) Lee, C.; Yang, W.; Parr, R. G. Development of the Colle-Salvetti correlation-energy formula into a functional of the electron density. *Physical Review B* **1988**, *37* (2), 785-789. DOI: 10.1103/PhysRevB.37.785.

(10) Clark, T.; Chandrasekhar, J.; Spitznagel, G. W.; Schleyer, P. V. R. Efficient diffuse function-augmented basis sets for anion calculations. III. The 3-21+G basis set for first-row elements, Li-F. *J. Comput. Chem.* **1983**, *4*, 294-301.

(11) Ditchfield, R.; Hehre, W. J.; Pople, J. A. Self-Consistent Molecular-Orbital Methods. IX. An Extended Gaussian-Type Basis for Molecular-Orbital Studies of Organic Molecules. *The Journal of Chemical Physics* **1971**, *54* (2), 724-728. DOI: 10.1063/1.1674902.

(12) Hehre, W. J.; Ditchfield, R.; Pople, J. A. Self—Consistent Molecular Orbital Methods. XII. Further Extensions of Gaussian—Type Basis Sets for Use in Molecular Orbital Studies of Organic Molecules. *The Journal of Chemical Physics* **1972**, *56* (5), 2257-2261. DOI: 10.1063/1.1677527.

(13) Feller, D. The role of databases in support of computational chemistry calculations. *Journal of Computational Chemistry* **1996**, *17* (13), 1571-1586.

(14) Hariharan, P. C.; Pople, J. A. The Influence of Polarization Functions on Molecular Orbital Hydrogenation Energies. *Theoretica chimica acta* **1973**, *28*, 213-222.

(15) Cossi, M.; Rega, N.; Scalmani, G.; Barone, V. Energies, structures, and electronic properties of molecules in solution with the C-PCM solvation model. *Journal of Computational Chemistry* **2003**, *24* (6), 669-681. DOI: 10.1002/jcc.10189.

(16) Klamt, A.; Schüürmann, G. COSMO: a new approach to dielectric screening in solvents with explicit expressions for the screening energy and its gradient. *J. Chem. Soc., Perkin Trans. 2* **1993**, 799-805. DOI: 10.1039/p29930000799.

(17) Cossi, M.; Barone, V. Quantum calculation of molecular energies and energy gradients in solution by a conductor solvent. *Journal of Physical Chemistry A,* **1998**, *102*, 1995-2001.

(18) Pritchard, B. P.; Altarawy, D.; Didier, B.; Gibsom, T. D.; Windus, T. L. A New Basis Set Exchange: An Open, Up-to-date Resource for the Molecular Sciences Community. *J. Chem. Inf. Model.* **2019**, *59*, 4814-4820. DOI: 10.1021/acs.jcim.9b00725.
